# Computational parametric mapping of functional neuroimaging data

**DOI:** 10.1162/IMAG.a.1130

**Published:** 2026-03-04

**Authors:** Simon R. Steinkamp, Iyadh Chaker, Felix Hubert, David Meder, Oliver J. Hulme

**Affiliations:** Danish Research Centre for Magnetic Resonance, Department of Radiology and Nuclear Medicine, Copenhagen University Hospital Amager and Hvidovre, Copenhagen, Denmark; Department of Physics, University of Trento, Trento, Italy; Department of Basic Neuroscience, University of Geneva, Geneva, Switzerland; London Mathematical Laboratory, London, United Kingdom; Department of Psychology, University of Copenhagen, Copenhagen, Denmark

**Keywords:** functional neuroimaging, variational Bayes, computational modeling, population receptive field, cognitive modeling, topographic mapping

## Abstract

Elucidating the neural basis of cognition requires theoretical models of cognition to constrain the modeling of neural data. A prevalent strategy in functional neuroimaging is to regress the latent variables of cognitive models onto neural data. Though widely used, this approach restricts the mapping of computational variables to single parameter values. We introduce computational parametric mapping (CPM), which builds on and generalizes the Bayesian population receptive field framework. CPM offers three main advances for cognitive computational modeling. First, it allows the fitting of cognitive models directly to neuroimaging data. Second, it allows for voxelwise or regionwise mapping of parameters of cognitive computational models onto the brain, thus making the topographic mapping methods prevalent in the sensory sciences available to the cognitive computational neuroscientist. Finally, it is efficient enough to make voxelwise mapping over large regions of interest feasible. Here, we illustrate how CPM can be used to fit reinforcement-learning algorithms to synthetic and real data.

## 1 Introduction

Many methods have been developed to relate behavioral and functional magnetic resonance imaging (fMRI) data (Cohen et al., 2017). One approach in cognitive and computational neuroscience is to incorporate cognitive models to localize brain regions that perform specific computations. There exists a diversity of approaches bringing together brain activity and cognitive modeling (Turner et al., 2017). One widely used approach is called model-based fMRI, where latent variables of cognitive computational models are regressed onto fMRI data, by incorporating them as parametric modulators in a general linear model (GLM) (O’Doherty et al., 2007).

This approach, for example, has been widely used in the field of reward neuroscience, where reinforcement learning algorithms provide models of observable behavior (Niv, 2009) that allow inference about latent states such as value estimations or the surprise about an outcome (reward prediction errors, RPEs) where the latter have been shown to be predictive of midbrain dopaminergic signaling (Schultz et al., 1997). Reinforcement learning algorithms, like other cognitive models, often have multiple parameters whose values need to be determined before the latent states and observables can be generated or inferred. In reinforcement learning, a core parameter is the learning rate α, which governs the degree to which new information impacts one’s expectation about future outcomes. In some tasks, such as classical conditioning tasks, the learning rate parameters were fixed a priori by the modeler (O’Doherty et al., 2003; Seymour et al., 2004). In other cases, behavioral responses such as choices can be used to infer model parameters from behavioral data (Niv et al., 2012), which has the advantage that the individual behavior of the learning process is also incorporated into the neural model. Fitting models to behavior first, then incorporating the associated latent variables into a model of neural activity, has thus become a common strategy in neuroimaging research.

This approach has now been used for over two decades and has generated valuable insights into how reward learning might be implemented in human and animal brains. However, new insights into how dopamine neurons signal RPEs pose challenges to the currently used approach. While it was originally thought that dopamine responses are typically homogeneous in their properties, a recent re-analysis of single-cell responses in the mouse midbrain shows a systematic diversity (Dabney et al., 2020). The recorded cells showed asymmetric firing patterns, responding with different sensitivities to better-than-expected versus worse-than-expected outcomes. Different dopamine neurons, thus, appear to have different degrees of “optimism” about upcoming future rewards. Algorithmically, this firing pattern is reflected in different learning rates for value updating upon better-than-expected ($\alpha^{+}$) and worse-than-expected outcomes ($\alpha^{-}$).

If there were to be a diversity of neural responses where different voxels generate RPEs with different parameter values for $\alpha^{+}$ and $\alpha^{-}$, this would not be revealed with the current modeling approaches. This is because fitting the model to behavior constrains the subsequent regression onto functional neuroimaging data to a single set of parameter values.

Mapping diverse parameter estimates onto the brain is not straightforward. Meder and colleagues attempted to do this with an ensemble of general linear models, each containing choice value regressors generated from a range of different learning rate parameter values (Meder et al., 2017). For each voxel, the learning rate of the choice value regressor best fitting to the voxel’s BOLD response was color-coded, generating topographic maps of learning rates over the anterior cingulate and inferior parietal cortex. Here, we propose a more direct estimation method, computational parametric mapping (CPM), which is inspired by and builds upon receptive field mapping techniques from the sensory domain. Kumar and Penny (2014) developed a method to estimate parameters of non-linear functions from fMRI data to model the receptive field structure of neuronal populations in auditory cortices. This method leverages the variational free energy framework (Friston et al., 2007), and similar to other methods (c.f. Friston et al., 2003) it infers the parameters that best map experimental inputs to the observed fMRI signal via convolution of a neural response and a hemodynamic response function. This method was subsequently adapted and applied to model population receptive fields in visual cortices and released as the BayespRF toolbox https://github.com/pzeidman/BayespRF (Zeidman et al., 2018) for the Statistical Parametric Mapping (SPM) package (www.fil.ion.ucl.ac.uk/spm) and hence referred to as the PRF approach.

Here, we have forked the BayespRF toolbox to generalize it so that it now estimates a receptive field over parameter spaces of cognitive models, rather than sensory (e.g., retinotopic or tonotopic) space. Even though it parallels the original concept, it is strictly speaking no longer a receptive field since it is not a field that defines receptivity to a part of stimulus space. We, hence, refer to these receptive fields more generically as population fields. In practice, this means we find the weighted average over a discretised parameter space (e.g., learning rates) that best describes the observed neural signal. This is effectively optimizing the parameters of model-based fMRI based directly on the neural data, independently of observed behavior.

We use the approach of estimating population fields over the parameter space of cognitive models for the same neurobiological reason it is used in sensory models for population receptive fields. A voxel’s BOLD response is the result of activity from tens of thousands of neurons. To model this, we approximate the combined response of these neurons as a weighted average over the parameter space of the cognitive model. This method is designed to reflect the fact that the response of the neuronal ensemble within the voxel is best represented by a distribution across the parameter space. By estimating a field over this space, we can capture the uncertainty inherent in the distribution of neuronal responses, thus accounting for the variability in the population of neurons that contribute to the voxel’s BOLD signal.

Adopting such a modeling framework to cognitive models has several advantages: First, we can fit neural response functions directly to neural data, widening the scope for modeling neural responses that are more diverse than can be captured with a single set of parameter values. Second, due to its Bayesian nature, we can derive the model evidence of different model classes and submit them to model comparisons. Third, by including a hemodynamic model as an observation model, the framework can accommodate regional neurovascular variations. Finally, by optimizing SPM’s integration algorithm and leveraging the possibility to precompute signals, this method is now efficient enough to be applied voxelwise over swathes of cortex, or indeed to many regions of interest.

In this paper, we lay out CPM in detail and demonstrate its functionality in the context of reinforcement learning. This is done for simulated data, which we use to assess parameter and model recovery, and in a proof-of-concept analysis applied to a real dataset. Though explained in the context of reinforcement and reward learning, its functionality is general to almost any cognitive model.

## 2 Computational Parametric Mapping

**Fig. 1.**
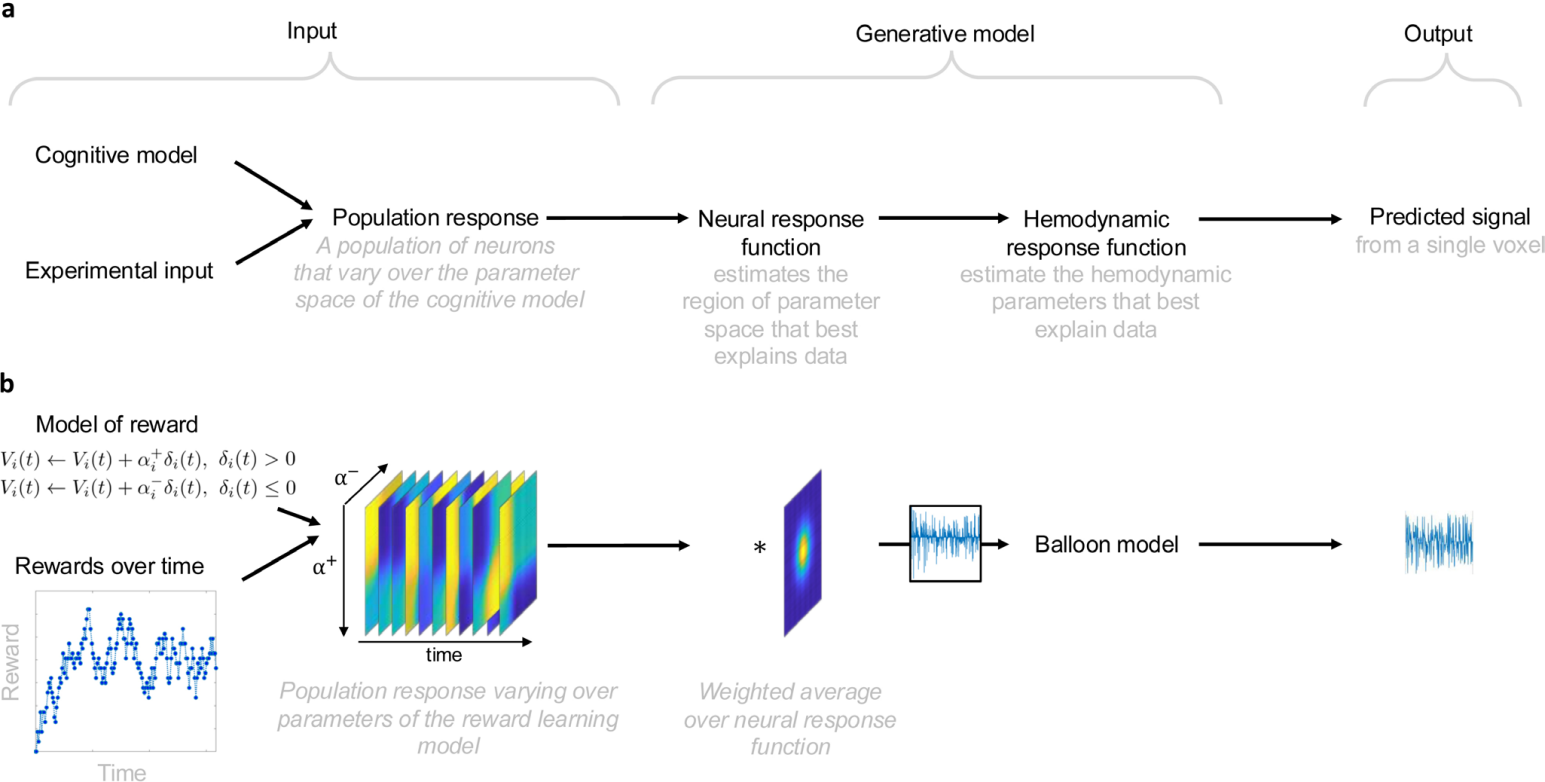
Schematic overview of computational parametric mapping. (a) Schematic shows the relationship between the inputs, the generative model, and the predicted signal. (b) Shows an example of this in which a model of reward learning is applied to a simple reward experiment. Here, the population response is a 3-dimensional matrix, where the 2-D sheets are the response of the hypothetical neurons in the population that differ according to the parameters of the reward model (two different learning rates, in the distributional reinforcement learning model), and the third dimension is time. This population response is weighted by the population field, generating a weighted average across the 2-dimensional parameter space. This, in turn, generates a 1-dimensional neural signal that inputs to the hemodynamic response function, which here is the balloon model, which has multiple parameters based on a mechanistic model of the hemodynamic response. The output of the hemodynamic model is the predicted fMRI signal time series to be used for optimising model evidence of the overall generative model.

In the following, we will describe the CPM framework in its general structure (Fig. 1a & 1b).

### 2.1 Inputs

In the original method by Zeidman et al. (2018) (an adapted version of Kumar & Penny, 2014) used for retinotopic mapping, the generative model consists of two components: 1. the neural response function, which is a population receptive field model mapping the experimental input to neural responses and 2. the hemodynamic response function, mapping the neural response function to the observable BOLD response of fMRI. In CPM, we do something similar; instead of finding the location and spread of a receptive field over a visual input, we aim at inferring the location and size of the population field over the parameter space of the cognitive model.

While visual stimuli already afford a concrete instantiation of the parameter values for the model (e.g., the x-y location of a bright stimulus in the visual field), we need an extra step for our method to build the space we wish to map into anatomical space. First, we need to define the free parameters Θ of the cognitive model of interest. We discretize Θ into the parameter grid P, by defining for each parameter $p^{k}\in \Theta$ their lower and upper bounds $(p_{min}^{k},p_{max}^{k}$), as well as a step-size ($r^{k}$) or resolution ($n^{k}$)—see Supplementary Materials A.2 for a detailed discussion of the discretization procedure. Using the points in the discrete parameter space P, we evaluate the computational model S for experimental inputs $x_{t}$ and obtain a time series $S_{t}$ over the parameter grid. Because the cognitive model S is only dependent on the experimental input, we can compute $S_{t}$ before estimating the generative model, thus moving some computational overhead outside the model inference step. However, estimating $S_{t}$ in this way might still be intractable, depending on the dimension and granularity of the grid, as well as the computational complexity of the cognitive model S.

**Fig. 2.**
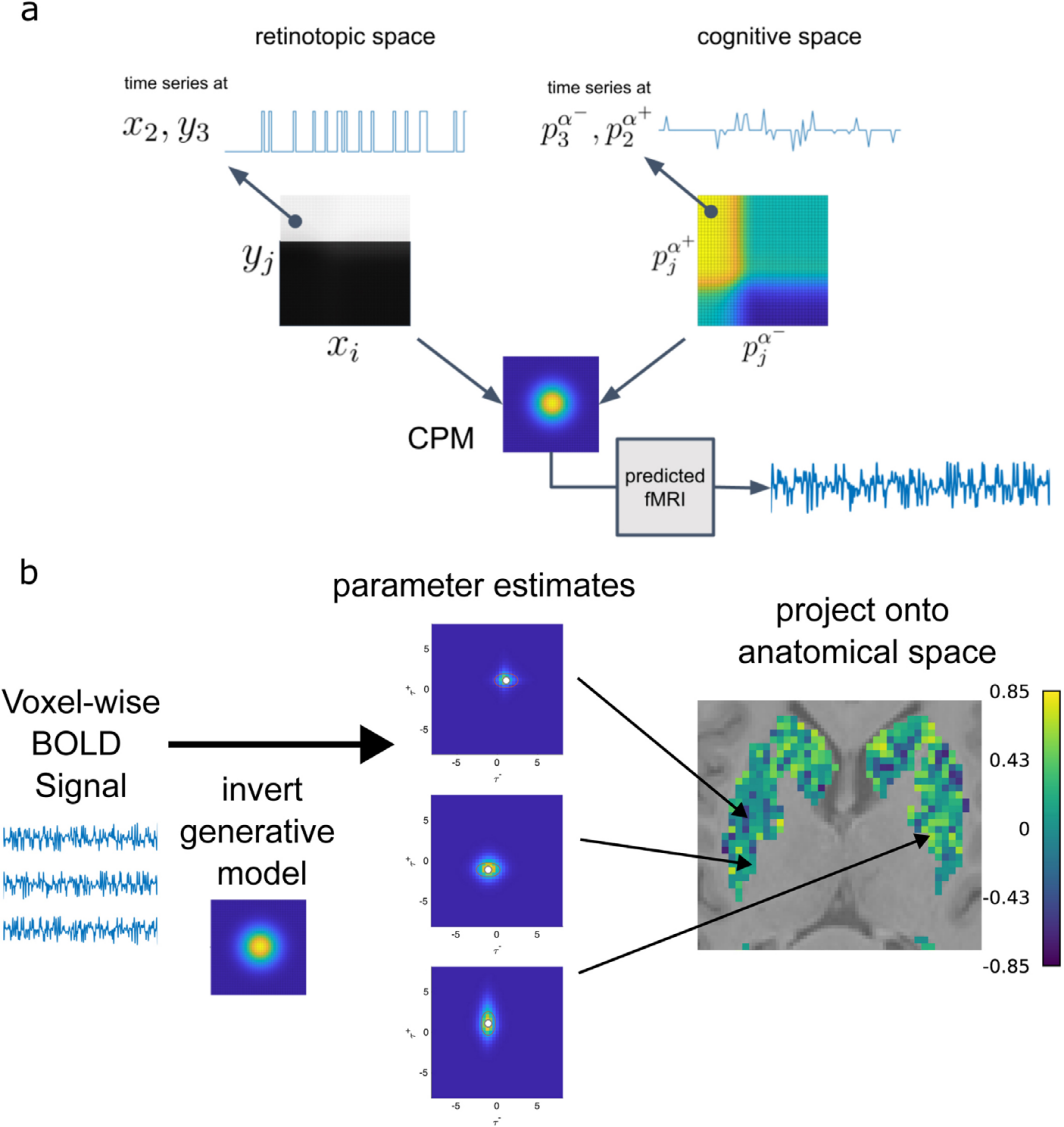
Equivalence between mapping retinotopic and cognitive spaces using CPM. (a) This figure highlights the equivalence between a time series of pixel intensities at point ($x_{i}$, $y_{j}$) in retinotopic space, and the time series of a latent computational variable within a cognitive space at point ($\alpha^{-}$, $\alpha^{+}$). The population field is then applied to the discrete parameter space P, and the bold response is estimated. (b) Here, we illustrate the workflow for topographic mapping. The voxelwise BOLD signal is extracted from a region of interest and subjected to CPM. After model inversion, we can then map the parameter estimates back onto the brain. Here, for example, exemplifying the learning rate asymmetry ($\alpha^{+}/\;(\alpha^{+}+\alpha^{-})$ ) in the striatum. The plots are meant to be purely illustrative and use simulated / random data.

In Figure 2, we draw a direct parallel to retinotopic mapping by considering a cognitive model with two parameters $\alpha^{-}$ and $\alpha^{+}$. It is important to note that the main difference between retinotopic space and the cognitive space in this example is that the former directly represents the visual stimulus signal, while the latter requires the precomputation of time series of latent computational variables that express themselves over the parameter space.

### 2.2 Neural response function

As in the pRF approach, we use a population field model to map $S_{t}$ to the neural signal $z_{t}$. In our example, this population field model is a normalized multivariate normal distribution N, meaning that $z_{t}$ is a weighted average over $S_{t}$. The neural response function $z_{t}$ is, thus, characterized by the moments $\mu_{p^{k}},\sigma_{p^{k}}$ representing location and spread for each $p^{k}\in \Theta$ . We also include a scaling parameter β, that controls the magnitude of the neural signal (“neuronal efficiency” (Kumar & Penny, 2014)). In practice, this parameter controls how much the neuronal signal contributes to the fitted BOLD response. For example, a very small β value indicates that the neuronal signal only contributes minimally to the BOLD activity in this voxel. Note that the magnitude of β depends on the scaling of S:

$$z_{t}=\beta \sum_{p_{i}\in P}S_{t}(p_{i})\cdot N(p_{i}|\theta_{n}),$$ (1)

where $\theta_{n}$ describes all the parameters of the population field model $\left\{\mu_{p^{1}},\sigma_{p^{1}}...\mu_{p^{n}},\sigma_{p^{n}},\right\}$ , which is denoted by a normal distribution (N) in our case.

### 2.3 Hemodynamic response function

The default hemodynamic model in the pRF toolbox is an extended balloon model (Buxton et al., 1998), which we also use for CPM. Most parameters in this model are fixed, based on empirical measurements (specific to the field strength of the MRI scanner), leaving three free parameters ($\theta_{h}$) to be estimated: the transit time v, the rate of signal decay κ, and the ratio of the intravascular to extravascular signal ℰ.

### 2.4 Model estimation via variational Bayes

Fitting generative models to data in this context depends on jointly fitting both neuronal and hemodynamic response functions. This process is repeated independently for each voxel. The posterior distribution of free parameters $\theta =(\theta_{n},\theta_{h})$ is found by fitting the generative model to the measured BOLD signals using variational Bayes. For more details, see Friston et al. (2007) and Zeidman et al. (2023). To summarize, let m be a generative model of fMRI data y, defined by the likelihood function p(y|θ,m) and the prior density p(θ|m) on model parameters θ. Variational Bayes (VB) is an iterative scheme that indirectly optimizes an approximation to both the model evidence p(y|m) and the posterior density p(θ|y,m) . The key to this optimization is to decompose the log model evidence into

$$log\;p(y|m)=F(q)+D_{KL}(q(\theta )||p((\theta |y,m)),$$ (2)

$$F(q,y)=log\;p(y|m)-D_{KL}(q(\theta )||p(\theta |y,m)),$$ (3)

where $D_{KL}$ is the Kullback-Leibler divergence, q is an approximate distribution, and F is the variational free energy. By maximizing F(q) , we are minimizing the divergence between the posterior distribution p(θ|y,m) and the approximate distribution q(θ) . This optimization is done by gradient ascent. Note that in this context, F refers to the negative variational free energy, which is maximized to achieve a better approximation of the model evidence; this is equivalent to minimizing the original free energy, thereby reducing the discrepancy between the approximate posterior and the true posterior. The exact inference scheme here is variational Laplace, which uses simplifying assumptions on the approximate distribution q(θ) , which renders the variational Bayes algorithm computationally and statistically efficient (Friston et al., 2007). One of these assumptions—the Laplace approximation—requires the model’s priors to be Gaussian. Given two concurrent models $m_{1}$ and $m_{2}$, the ratio of the minimized free energies for two models $F_{1}^{*}$ and $F_{2}^{*}$ approximates the Bayes Factor between those same two models:

$$B_{12}=\frac{p(y|m_{1})}{p(y|m_{2})}\approx exp(F_{1}^{*}-F_{2}^{*}).$$ (4)

This allows us to perform Bayesian model comparison and selection by choosing the model with the strongest model evidence.

### 2.5 Latent parameter transforms

Variational Laplace requires that parameters are expressed in terms of Gaussian distributions (not to be confused with the population field, which is also Gaussian). This means that we need to transform the latent parameters of the model so that they are Gaussian, therefore not extending beyond the bounds of the parameter space. Here, we use a uniform distribution over the parameter space. This means the following transformations are applied to the latent Gaussian parameters of the variational Laplace inference scheme:

$$l_{\mu }∼N(0,\;1)\Rightarrow \mu ∼U(\mu_{min},\mu_{max})$$ (5)

$$l_{\sigma }∼N(0,\;1)\Rightarrow \sigma ∼U(\sigma_{min},\sigma_{max})$$ (6)

$$\mu =(\mu_{max}-\mu_{min})*ncdf(l_{\mu })+\mu_{min}$$ (7)

$$\sigma =(\sigma_{max}-\sigma_{min})*ncdf(l_{\sigma })+\sigma_{min}$$ (8)

where ncdf is the cumulative density function for the univariate normal distribution. $\mu_{min}$ and $\mu_{max}$ are the boundaries of the location parameter, and $\sigma_{min},\sigma_{max}$ are the limits over the spread of the population field. The exact values are dependent on the discretization and are calculated automatically (see Supplementary Materials A). Similarly to the HRF parameters ($\theta_{h}$), the scaling parameter β is constrained to be positive, using a normal latent parameter $l_{\beta }$ and the transformation $\beta =exp(l_{\beta })$ .

### 2.6 Minimizing compute

The Bayesian inference procedure, as implemented in the BayespRF toolbox, has a high computational cost of model estimation when it is applied to numerous voxels or volumes of interest. As the number of voxels often exceeds the number of compute cores available, parallelization alone is not sufficient. In CPM, we improve the computation time by restricting the execution of the SPM12 integration function, spm_int, which takes up to 80% of the computation time in its standard implementation. This numerical integration generates the BOLD signal from a neuronal signal $z_{t}$ and a hemodynamic model, solving the differential equations representing the hemodynamics at each time step t. In our example case, the neuronal signal is sparse, only taking on values different from 0 at stimulus onsets (ca. 4% of the oversampled signal). The spm_int function unnecessarily calculates the solution for the differential equations for every time step, even for all the time steps where the neuronal signal equals 0. We modify this function to store the solution for $z_{t}=0$ , solving the differential equation only for non-zero values. By testing this on our model, we get a substantive speed-up compared to the normal execution of spm_int. Of course, this speed-up is dependent on the sparsity of the neuronal signal and the hardware used. To demonstrate the usefulness of this simple modification, we simulate neuronal inputs with varying sparsity and plot the speed-up compared to the original spm_int (Supplementary Materials E, Fig. S7). The presented modification has no tradeoffs and results in the same output as normal execution. This change could improve computational time for many other SPM methods, such as DCM. The updated function can be found in Supplementary Materials E.

## 3 Simulation Study

To test the feasibility and robustness of our method, we first conducted a simulation study, which we use to illustrate the CPM method.

**Fig. 3.**
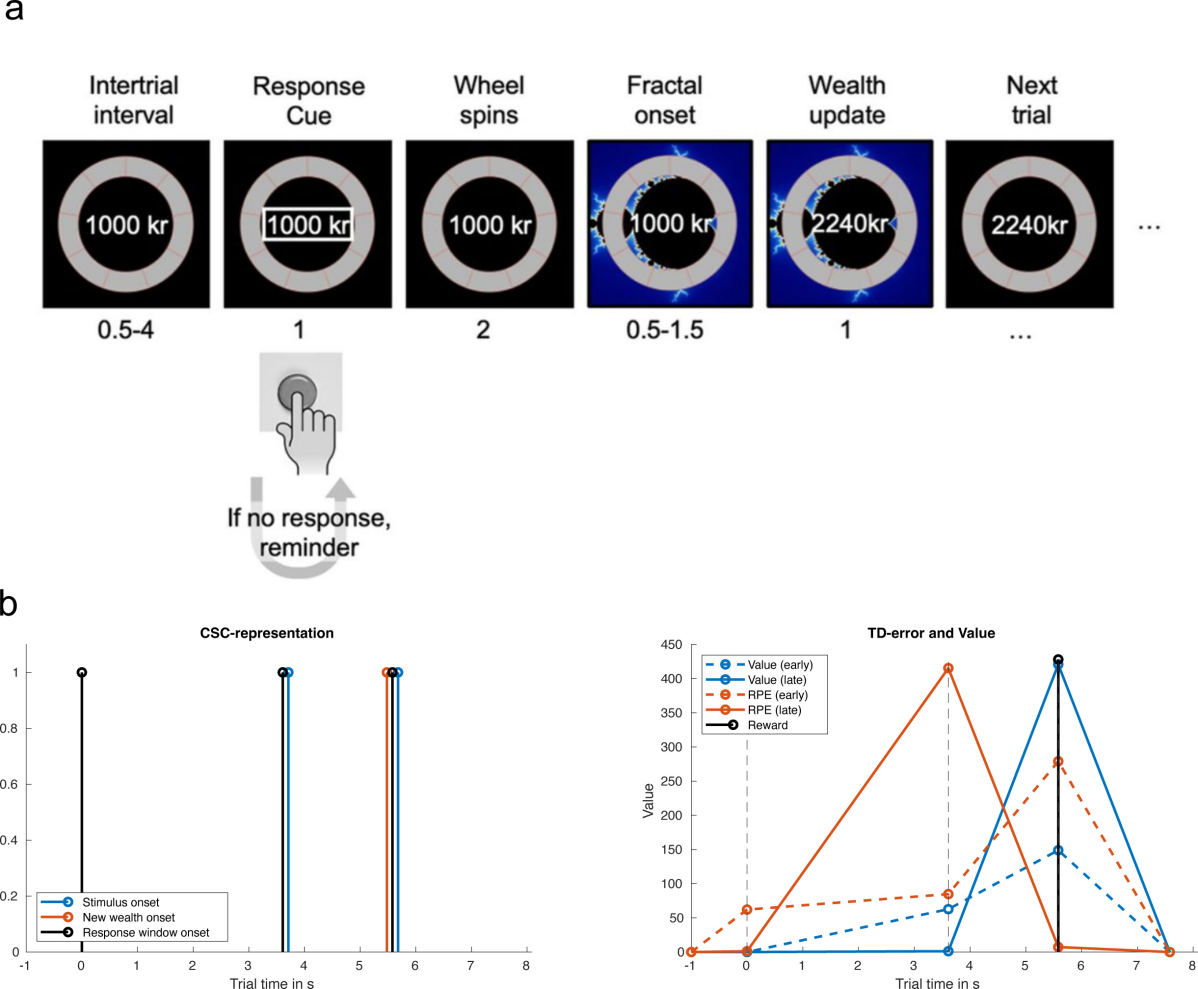
(a) Timeline of the experiment. Participants see a response cue, and after pressing the button, the “wheel” on the screen starts spinning. Then the image is displayed, and the associated change in wealth is realized. Figure adapted from Meder et al. (2021), published under CC-BY 4.0 https://creativecommons.org/licenses/by/4.0/. (b) TD-learning representation. Left: Modeling of the onsets in the experiment as a complete serial compounding representation. Three timesteps are considered: the response cue onset, the onset, and identity of the stimulus and the onset of the reward (i.e., the wealth update). The model learns weights, i.e., a prediction for each of the steps in a single trial. Right: The learning process. Upon repeatedly learning the value of a stimulus, the RPE (orange lines) moves from reward onset in early trials (dashed lines) to stimulus onset in late trials (solid lines). However, the value V begins to more and more approximate the reward magnitude.

Our simulation builds upon an experiment involving a passive monetary reward learning task used in Meder et al. (2021). In the task (Fig. 3a), subjects are shown a series of nine images, each having a deterministic effect on their wealth (as depicted in the “Rewards over time” time-series on the left of Fig. 1b). They are to learn the reward value of each image via passive observation. An experimental run of the task consists of 168 trials, each comprising three separate events: the trial’s onset, the onset of the image, and the image’s effect on the participant’s wealth (wealth update). As our cognitive model, we use a reinforcement learning model to capture the reward learning process and simulate the BOLD response to the reward prediction error δ.

In an experimental setting, we might ask questions such as:

- Does our region of interest encode δ?
- What learning rates best characterize the encoded δ?
- Is a single learning rate sufficient to explain the data, or is a model with different learning rates for positive and negative δ better able to explain the data?
- What is the anatomical topography of learning rates across our region of interest?

For our simulation study, we will compare two versions of temporal difference (TD) learning models of reward, namely classical TD versus risk-sensitive TD (Niv et al., 2012) (see Supplementary Materials D, for simulations including a different model class).

### 3.1 Classical TD model

The algorithm uses serial compounding (Ludvig et al., 2012), that is, backpropagating reward prediction errors at trial onset, stimulus onset, and reward delivery to iteratively update value predictions of future rewards (Fig. 3b, left). The algorithm aims to incrementally learn an estimate of the true future reward ($V^{*}$), defined by the weights w and the state vector x:

$$V(t)=\sum_{i=1}^{n}w_{t}(i)x_{t}(i).$$ (9)

x here is a binary-valued representation of the environment’s current state, typically composed of temporally coarse-coded features that span the trial duration. At each time step, the weights ($w_{t}$) are updated using the TD-error ($\delta_{t}$), which is computed as follows:

$$\delta_{t}=r_{t}+\gamma V_{t}-V_{t-1},$$ (10)

where $V_{t}$ is the reward value expected at time t (Fig. 3b, right). The reward $r_{t}$ is defined as the change in wealth caused by the image, and γ is the temporal discount factor. The weights are then updated using the learning rate α and the eligibility trace $e_{t}$. The learning rate parameter α controls the degree to which reward expectations are updated by experience and controls the trade-off between the speed and accuracy of learning:

$$w_{t+1}=w_{t}+\alpha \delta_{t}e_{t}.$$ (11)

Finally, the eligibility trace is updated at each step, with its decay rate (λ) governing how the trace’s influence diminishes over time:

$$e_{t+1}=\gamma \lambda e_{t}+x_{t}.$$ (12)

### 3.2 Risk-sensitive TD model

The risk-sensitive implementation extends the TD learning model above to have different learning rates for positive and negative reward prediction errors (cf. Niv et al., 2012). For the TD-learning algorithm, this means that we replace the updating of the weights with the following formula:

$$w_{t+1}=\{\begin{array}{cc} w_{t}+\alpha^{-}\delta_{t}\epsilon_{t}, & \text{if}\;\delta_{t}\leq 0\\ w_{t}+\alpha^{+}\delta_{t}\epsilon_{t}, & \text{if}\;\delta_{t}>0. \end{array}$$ (13)

### 3.3 Linearizing the learning rate parameter space

Variational Laplace requires that parameters are expressed in terms of Gaussian distributions. This can be problematic for the estimation of truncated parameters, such as the learning rate, which is constrained to 0≤α≤1 and where values close to the boundaries can no longer be estimated with a Gaussian that would extend beyond that boundary. We, thus, introduce τ as a latent parameter of the learning rate, so that $\alpha =\frac{e^{\tau }}{1+e^{\tau }}$. This reparameterizes the learning rate using the latent variable τ and a sigmoid function to better linearize the parameter space. This approach is beneficial because it makes the parameter space more well-defined and easier to work with, both in terms of its probable (likely) and possible (theoretically allowed) values and visualization (see Supplementary Materials A.2).

### 3.4 Simulating data

To test the feasibility of our method, we conducted a simulation study to assess the model’s robustness to noise as well as the model’s and parameter identifiability (i.e., model and parameter recovery). Recovering both model structures and parameters from simulated data is not trivial because the ability to recover information about the generative process depends fundamentally on the experimental design. In many experimental designs, different generative processes can generate identical data, leading to difficulties in recovering the “true” model or parameter.

In our simulation study, we are interested in testing whether we can dissociate BOLD signals generated by risk-sensitive versus classical TD models. We generated data from several generative processes, corresponding to either model, over different parameters, which we will refer to as simulated “voxels”. We are both interested in recovering the location and the spread of the population field.

The classical TD model is defined by a single learning rate parameter τ. The population field is, thus, made of a single location $\mu_{\tau }$ and a single spread $\sigma_{\tau }$. We simulated data for four voxels made up by the parameter combinations of $\mu_{\tau }\in \{-1.0986,\;1.0986\}$ (corresponding to learning rates α∈{0.25, 0.75} in non-linearized space) and $\sigma_{\tau }\in \{0.4394,\;$ 0.7324} .

For the risk-sensitive TD process, we simulated 16 voxels, generating data with all combinations of the following parameter values: $\mu_{\tau^{-}}\in \{-1.0986,\;1.0986\}$ , $\mu_{\tau^{+}}\in \{-1.0986,\;1.0986\}$ , $\sigma_{\tau^{-}}\in \{0.4394,\;0.7324\}$ , $\sigma_{\tau^{+}}\in$ {0.4394, 0.7324} .

For all simulated voxels, we used β=exp(0.5) and prior values for the HRF parameters (i.e., $\theta_{h\tau }=0.3$ , $\theta_{h\kappa }=0.2$ , $\theta_{hg}=-0.5$ ). We then add Gaussian noise to the BOLD signal, with 0 mean and standard deviations {0.0076, 0.0240, 0.0603, 0.0956, 0.2402, 0.7594} , which roughly translate to signal-to-noise ratios of {20, 10, 2,  −2, −10, −20} , where SNR is defined as $10{\text{log}}_{10}\left(\frac{\sigma_{\text{signal}}^{2}}{\sigma_{\text{noise}}^{2}}\right)$ (Welvaert & Rosseel, 2013). This allowed us to assess parameter recovery across a feasible space of parameters. We simulated the data at a sampling rate with a repetition time (TR) of 0.592 s, the same TR as in the real data used in Meder et al. (2021).

We defined generative models to recover the parameters and processes of the simulation. Since locations in the population field are estimated as Gaussians with standard deviations, we define a range of *probable* parameter values for τ with $\mu_{min}=-4$ and $\mu_{max}=4$ (corresponding to α=0.018 and α=0.982 in non-linearized space). To estimate the Gaussian around these locations with a certain standard deviation σ, we needed to extend the *possible* parameter values beyond $\mu_{min}$ and $\mu_{max}$ (see Supplementary Materials A.2): For classical TD, we defined a 1-dimensional grid P over τ∈[−8.1259, 8.1259] (corresponding to α∈[0.0003, 0.9997] in non-linearized space), discretized over 41 evenly spaced points. The risk-sensitive TD model used a 2-dimensional grid P over $\tau^{-}\in [-8.1259,\;$  8.1259] and $\tau^{+}\in [-8.1259,\;8.1259]$ , discretized over 41 points along either dimension. We can now attempt to recover the population field parameters to assess the accuracy of the generative modeling and its robustness to measurement noise.

In our case, the δ signals for each set of parameters are normalized to the range (−1, 1) . Compressing the magnitude of the δ trajectories, thus, carries information about the shape of the learning process (i.e., the learning rate); however, it does not carry information about the absolute magnitude of δ, which can be vastly different for different parameter settings. Furthermore, we set the hyperparameters to γ=0.99 and λ=1.0 .

## 4 Simulation Results

### 4.1 Bayesian model selection

**Fig. 4.**
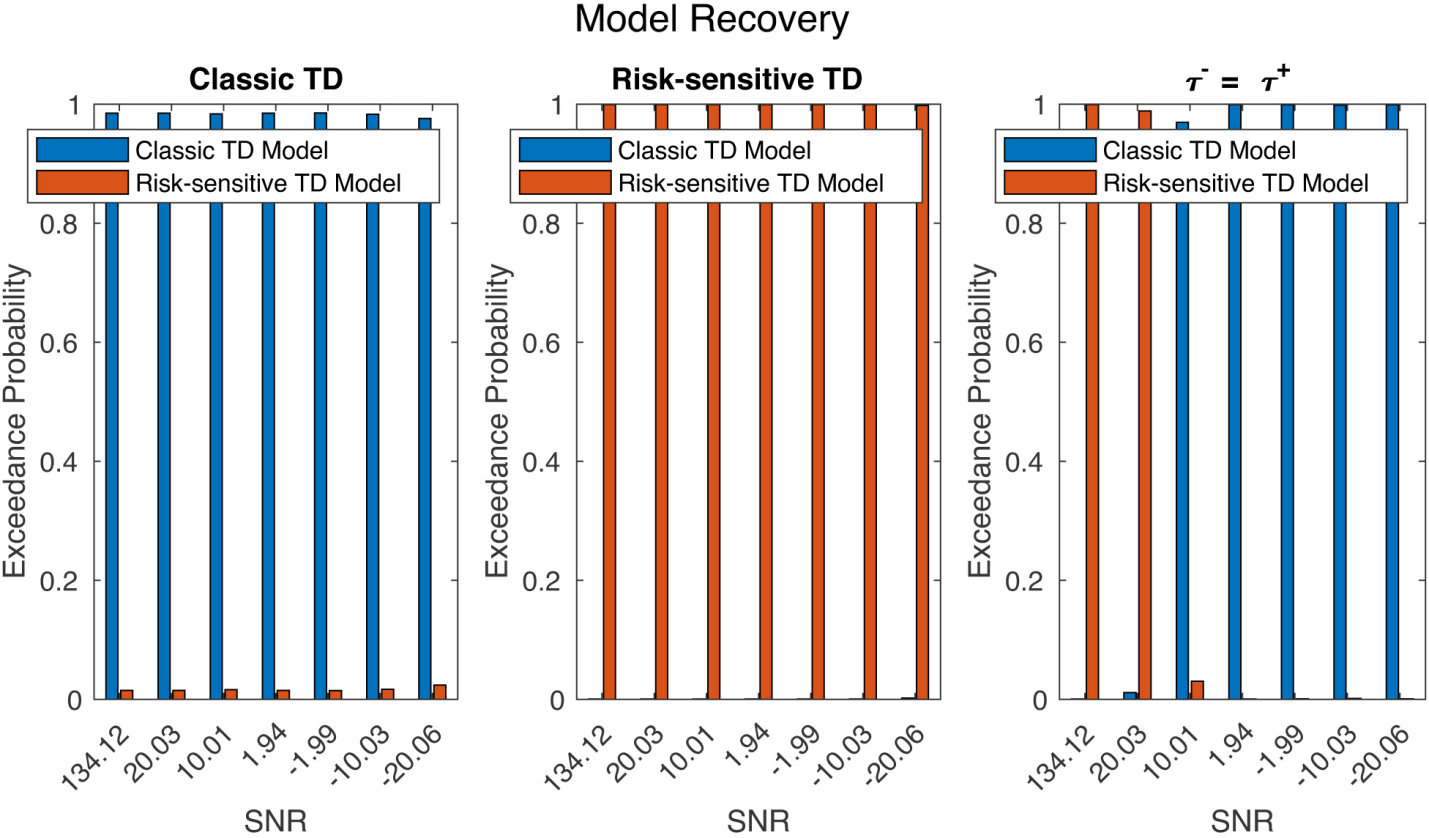
Bayesian model comparison for model recovery data. Bar graphs of exceedance probabilities for Bayesian Model Selection for the different data-generating processes. In the left plot, the data are generated by a classical TD process (averaged across all models with different parameter values for τ), and indeed the classical TD model outperforms all other models at all signal-to-noise ratios. In the middle plot, the data are generated by the risk-sensitive-TD process (averaged across all models with different parameter values for $\tau^{+}$ and $\tau^{-}$ except those where $\tau^{+}=\tau^{-}$), and likewise, the risk-sensitive-TD model outperforms all other models over all signal-to-noise ratios. Finally, in the right-hand plot, we show the special case where the risk-sensitive-TD process generates the data, but where it is set such that it is identical to a classical model. Here, the classical model outperforms the risk-sensitive model (in all cases but those where the data were generated with the highest SNR) because it makes the same predictions while also being more parsimonious. These plots illustrate the validity of the model selection procedures.

After inverting the generative models using data from each of the generative processes, we investigate in the first step if we can accurately recover the generative process, using Bayesian model selection implemented in the Variational Bayesian Analysis toolbox (Daunizeau et al., 2014). We performed three recovery tests: 1) Recovering the classical TD process, 2) recovering the risk-sensitive TD process, and 3) testing the model recovery for degenerate cases in which $\mu_{\tau^{+}}=\mu_{\tau^{-}}$. The last case is a degenerate case because although it is generated by a strictly risk- sensitive TD model, it generates the same data as a classical TD model, albeit with the flexibility of allowing different variances around $\mu_{\tau^{+}}$ and $\mu_{\tau^{-}}$. We can see, in Figure 4, that model recovery was successful for the generative processes of classical TD and risk-sensitive TD models. For case 3, however, the classical TD model has consistently the highest evidence, except for those models generated with the highest SNR, even though the generative model was based on a risk-sensitive TD model with varying variances. This is behaving as expected because the classical TD model is better at explaining the data by virtue of its lower complexity.

### 4.2 Parameter recovery

**Fig. 5.**
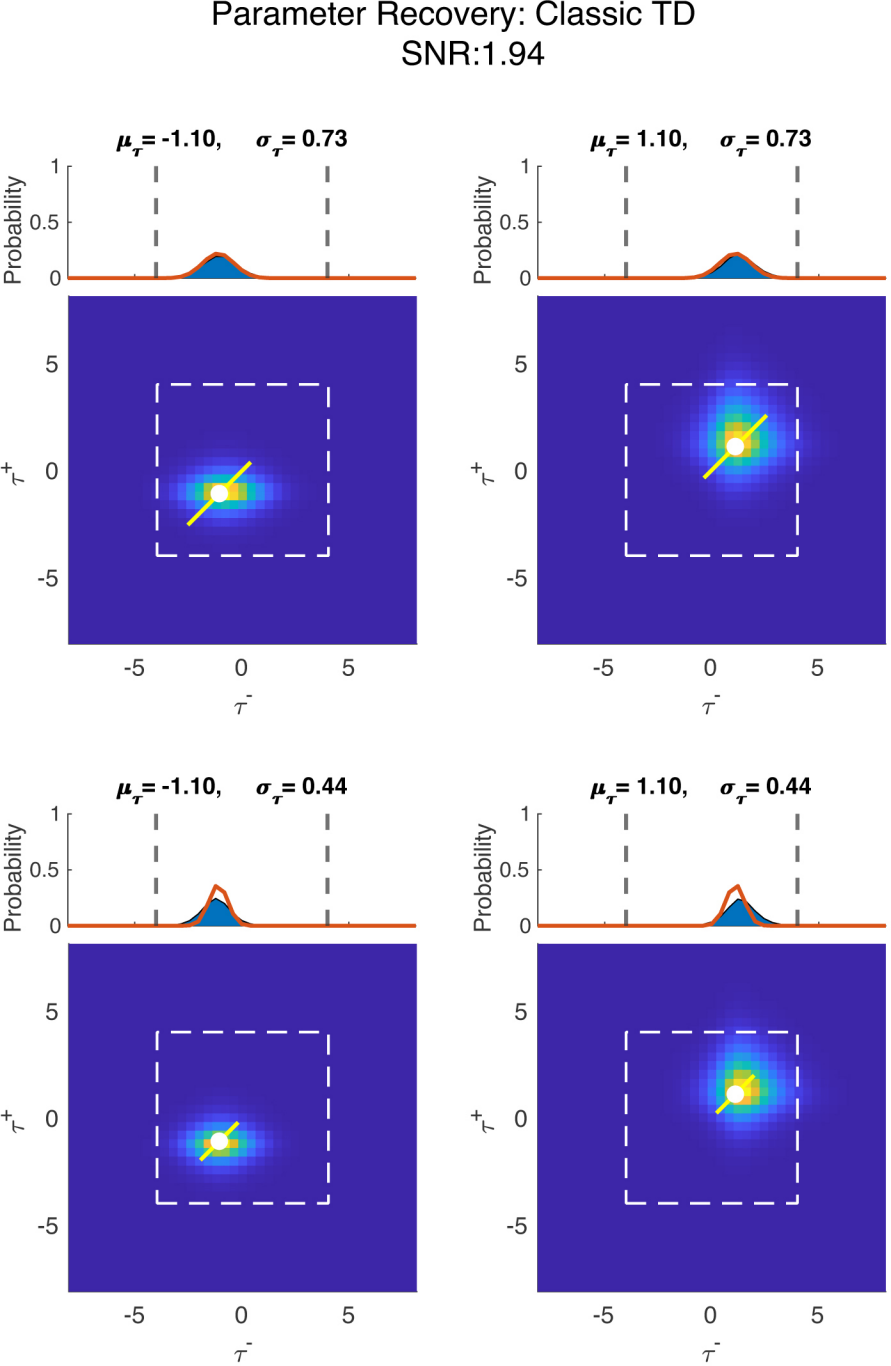
Parameter recovery plot for data generated from a classical TD generative process. The four panels represent the parameters tested. At the top of each panel, we see the 1-dimensional population field in orange, which was used in the generative process of the classical TD model; the blue area is the recovered field by the generative model. The dashed gray lines describe the probable parameter space (Supplementary Materials A.2, Fig. S2). Below is the recovered population field by the risk-sensitive TD generative model, where the parameters and standard deviation used for generating the data are indicated by the white dot and line. The dashed white square in the panel describes the probable parameter space.

**Fig. 6.**
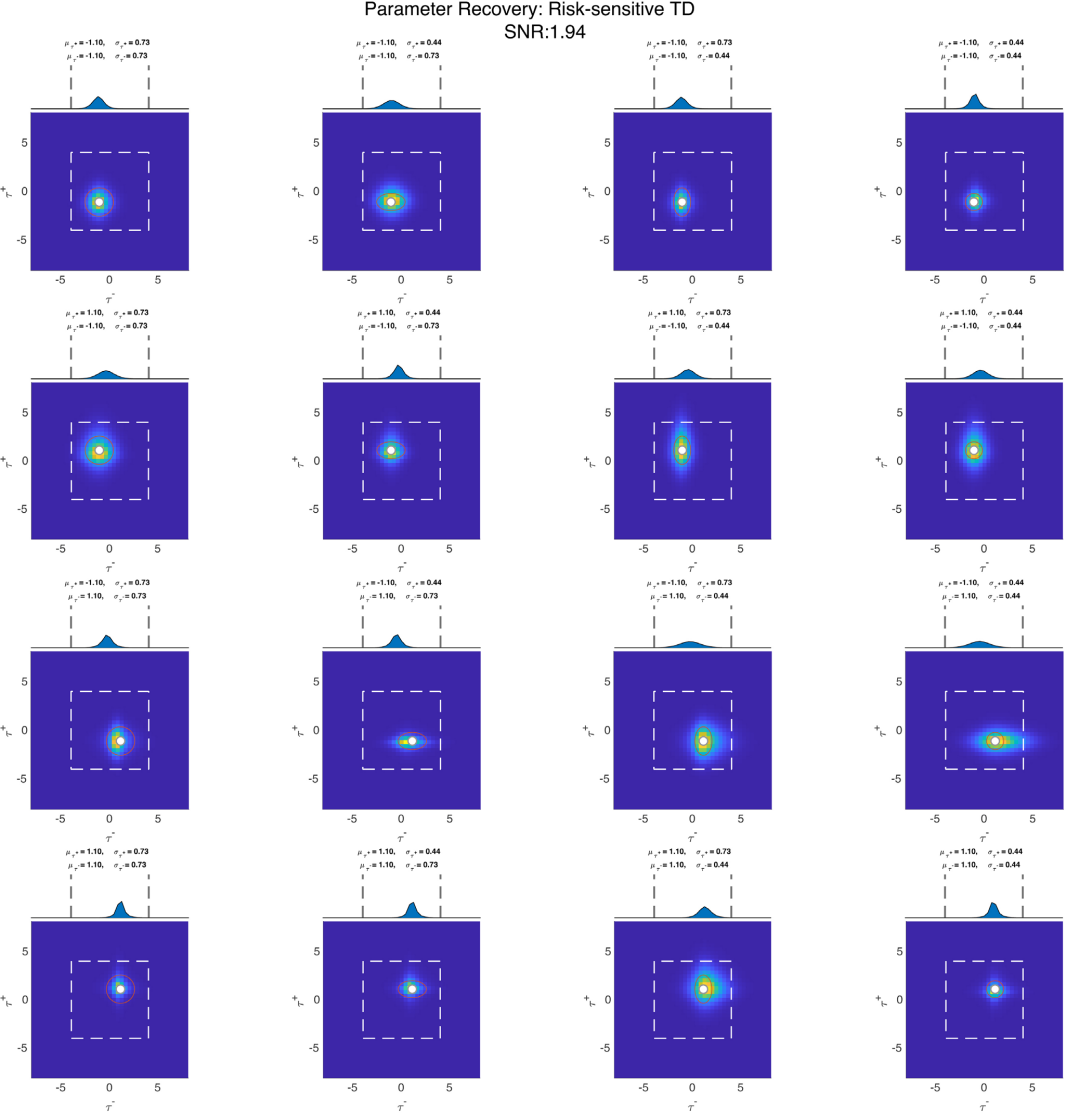
Parameter recovery plot for data generated by a risk-sensitive TD generative process. The 16 panels represent the different combinations of parameter values used for data generation. At the top of each panel, we see the blue area, which is the recovered field obtained by estimating the classical TD model. The dashed gray lines describe the probable parameter space. The subpanels below show the recovered population field obtained from the risk-sensitive TD generative model, where the field used by the generative process is indicated in red and the white dot indicates the location of the field. The dashed white square in the panel describes the probable parameter space.

Having generated the simulated voxels and recovered the models that generated them, we can now apply CPM to recover the parameters of the generative process. In Figures 5 and 6 are plots of the recovered population fields for data generated from classical and risk-sensitive TD models, respectively. We show data simulated with a realistic (with respect to neuroimaging data) SNR of ∼1.94 (SD of Gaussian noise = 0.0603). We make a couple of observations from these plots. First, we see that the recovery of the location parameters works generally quite well. There seems to be some slight bias toward higher values, especially when the τ values used for data generation are in the positive τ range. We further see that CPM has some issues in recovering the exact spread of the data-generating process. Typically, however, we do not anticipate inferring the spread of a process to be the primary inferential goal when mapping cognitive parameters.

**Fig. 7.**
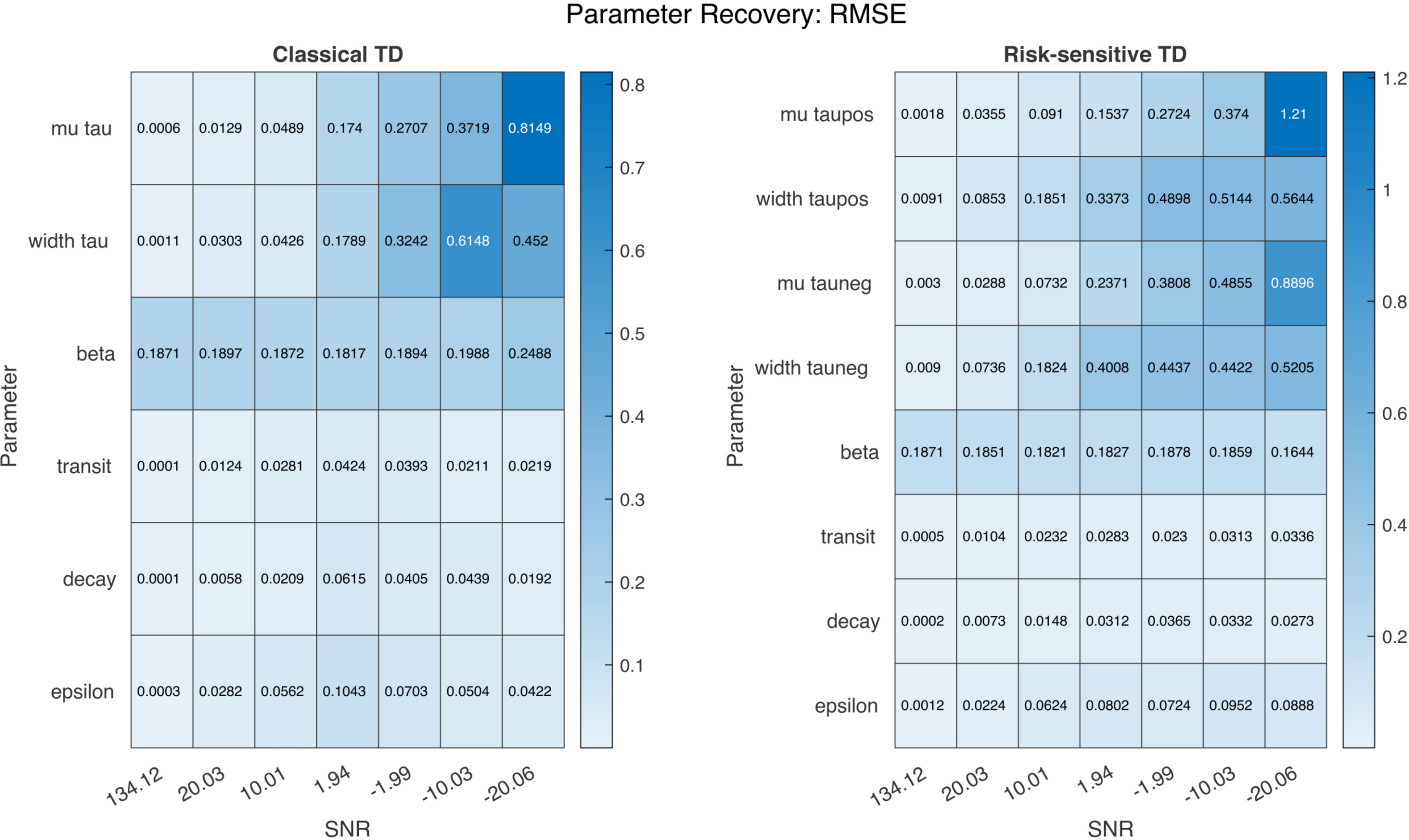
Estimation error for classical TD (left) and risk-sensitive TD (right). We display here the root mean square error (RMSE) of the parameter recovery separately, for the generative model and generative process of classical TD and similarly, risk-sensitive TD. Errors are calculated over all models at a given SNR. The population field parameters (including β) were transformed from latent to native space before the error was calculated.

Parameter recovery, especially the location parameters, is generally very accurate, with increasing error at higher noise levels. In the risk-sensitive TD scenario, there appears to be some bias for $\tau^{+}$ parameters toward higher values, with a somewhat higher error than the $\tau^{-}$ parameters, although this reverses at higher noise (Fig. 7). We also observe a relatively high error in the recovery of the scaling parameter β; these errors are relatively constant across noise.

**Fig. 8.**
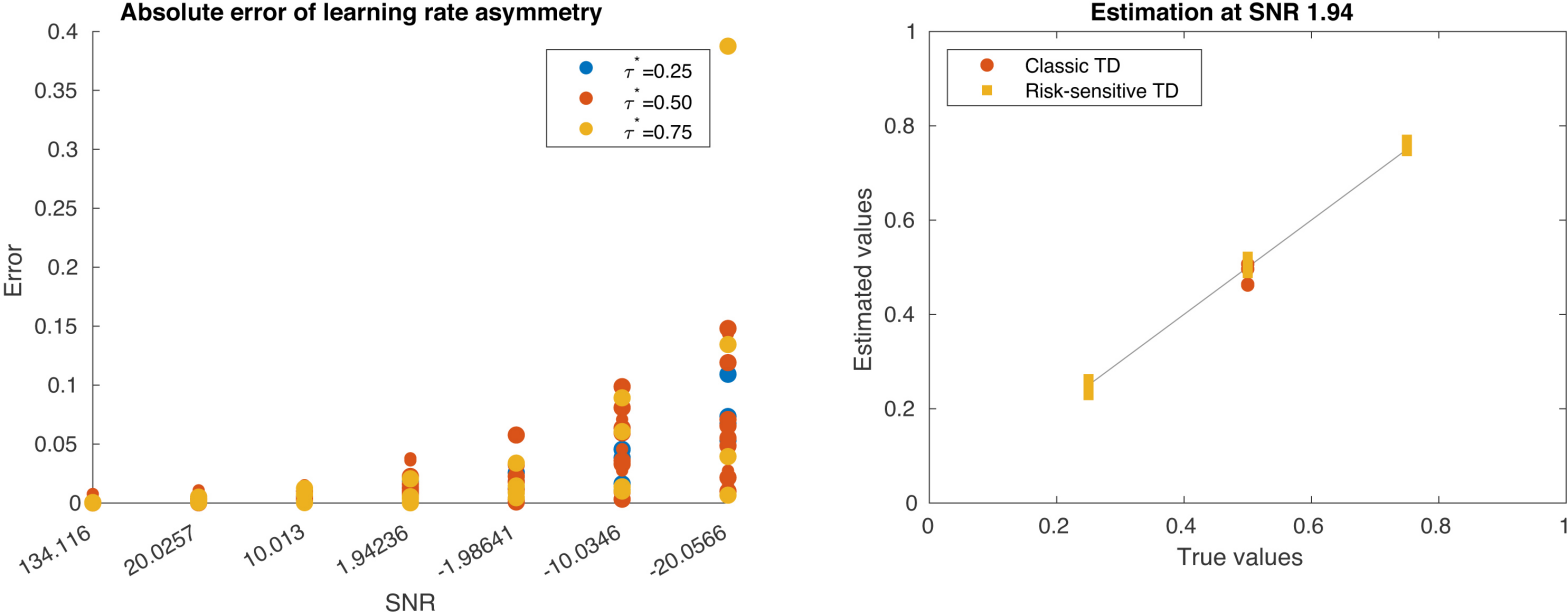
Recovery of the learning rate asymmetry (τ* ), which was calculated by transforming the model parameters of the risk-sensitive TD model $\tau^{-}$ and $\tau^{+}$ into learning rates ($\alpha^{+}$ and $\alpha^{-}$). On the left, the absolute error of the recovery of τ* is displayed, colored by the different asymmetry levels and for each SNR. On the right, the estimated values at an SNR of 1.94 are plotted against the true values, where color indicates if the values were recovered from a risk-sensitive or classical TD generative process.

For the recovery of risk-sensitive TD parameters, not only is the actual fidelity of parameters to be recovered important, but also the relationship between $\tau^{+}$ and $\tau^{-}$. For this, we introduce the definition used in Dabney et al. (2020), where they define a parameter $\tau *=\frac{\alpha^{+}}{\alpha^{+}+\alpha^{-}}$, which signifies the “optimism” of the model. Optimism represents the asymmetry between the learning rates of the positive and negative reward prediction errors. As this is a potential use case for CPM, we also plot the fidelity with which we can recover τ* from the data. As is to be expected from Figures 5 and 6, Figure 8 shows a generally good recovery of τ* , which slightly deteriorates at lower SNRs.

### 4.3 Posterior correlation

**Fig. 9.**
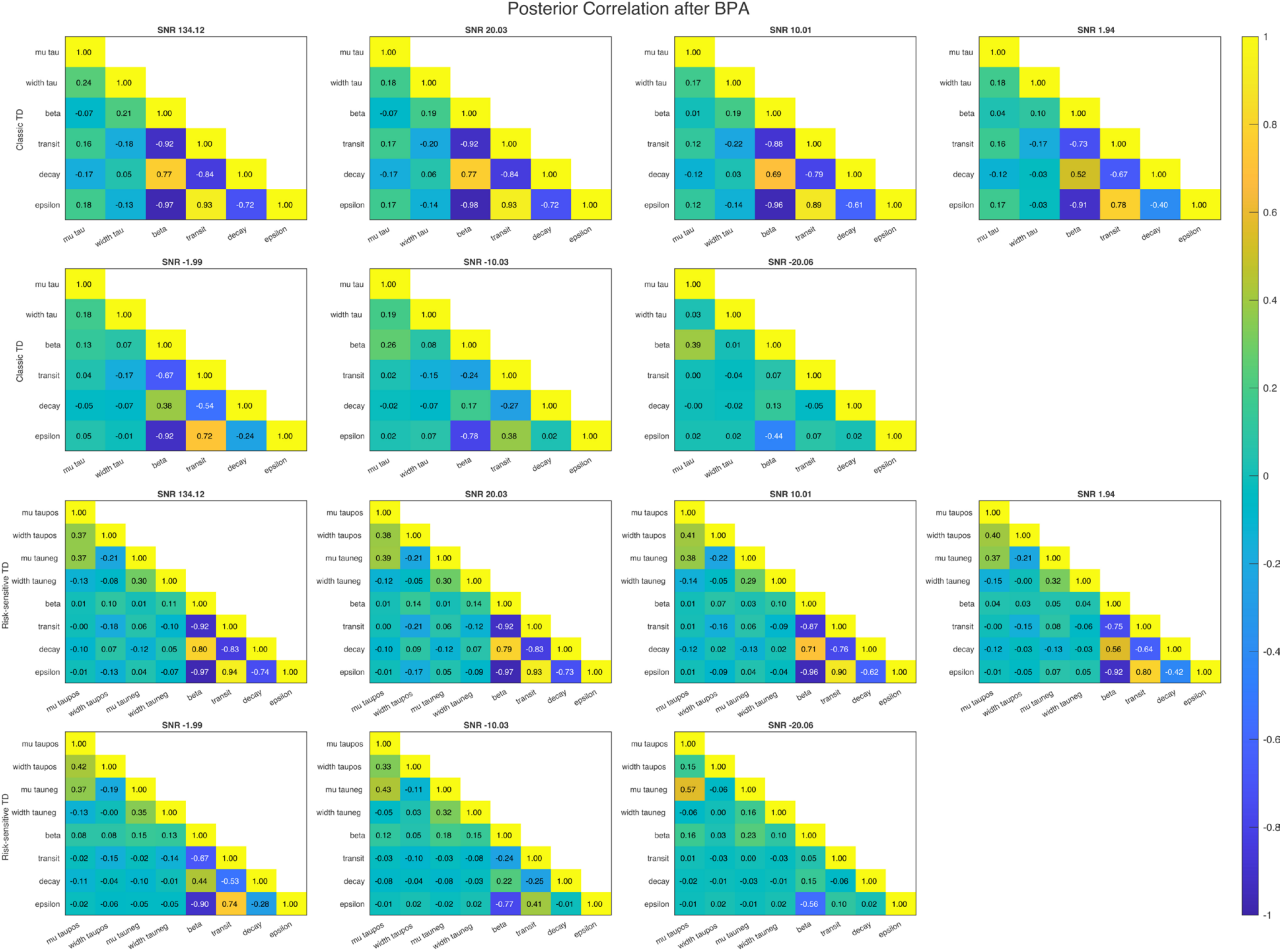
Posterior parameter correlation of classical TD (upper row) and risk-sensitive TD (lower row) models, estimated by calculating Bayesian Parameter Average and transforming the posterior covariance matrix into a correlation matrix. Each column represents one of the noise levels. Note that we use the latent parameters here (indicated by names like “lmu_tau”).

We further investigate the posterior correlation of the parameter estimates and the true parameters of the generative process by calculating the Bayesian Parameter Average over each of the 4 (or 16) candidate models and converting the posterior covariance matrix to correlation values. What is striking from looking at Figure 9 is that there is a very high correlation between the latent scaling parameter (β) and the transit time parameter (v) of the hemodynamic response function, as well as high correlation values between the different hemodynamic parameters. For our model, there is a high correlation between $\mu_{\tau^{+}}$ and $\mu_{\tau^{-}}$, due to the fact that in 50% of models these parameters actually have the same value. Removing parameters where $\tau^{+}=\tau^{-}$ leads, indeed, to lower correlations (Supplementary Materials A.1, Fig. S1).

### 4.4 Classical TD model fit

**Fig. 10.**
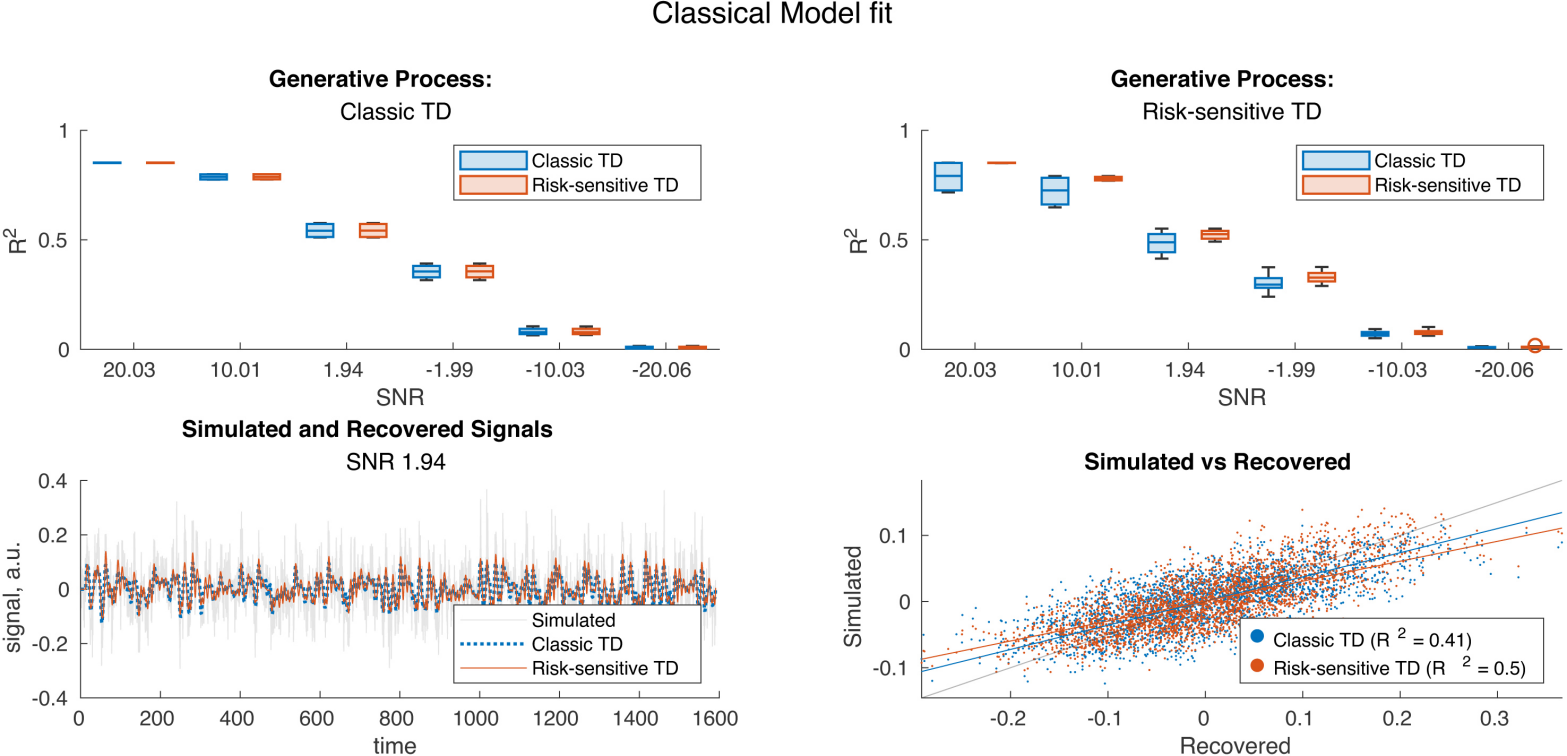
Overview of model fit statistics ($R^{2}$) estimated between the simulated and recovered data. In the upper left, we see bar plots of the model fit of classical TD (blue) and risk-sensitive TD (red) recovering a classical TD generative process. On the right, the same, but for a risk-sensitive TD process. The lower row is an example of the signal simulated by a risk-sensitive TD generative process with parameters ($\mu_{\tau^{-}}=1.0986,\;\;\mu_{\tau^{+}}=-1.0986,\;\sigma_{\tau^{-}}=0.4394,\;\sigma_{\tau^{+}}=0.7324$ ) and the recovery by a classical TD and risk-sensitive TD process. The lower right is a scatter plot of the data in the lower left, with least square fit lines and a gray fiducial indicating a perfect fit.

In a final step, we used the inverted models to simulate a BOLD signal and test it against the original BOLD signal. We see that there is generally a high fidelity between the two BOLD signals in terms of $R^{2}$ scores, regardless of the model used (Fig. 10). However, the model representing the true generative process has some edge over the other model.

## 5 Real Data

### 5.1 Ethics

There are no new data presented in this section, only simulated data and openly accessible data. The original ethics statement for the open dataset is available within Niv et al. (2012).

### 5.2 Methods

In a further analysis, we applied the classical TD and the risk-sensitive TD models to an openly accessible dataset from Niv et al. (2012), which was shared with Wilson and Niv (2015). The dataset provides averaged BOLD data from two regions of interest (right and left nucleus accumbens) from a risk-sensitive decision-making task. It also provides regressors with the time series of RPE values derived from a classic as well as a risk-sensitive TD learning model fitted to behavior, convolved with the canonical HRF function from SPM. Since the dataset only provides two BOLD time series, CPM cannot reveal an anatomical topography of learning rates across many voxels. The dataset does, however, allow us to compare the model fit from the classic model-based fMRI approach to the one returned by CPM. Furthermore, we can demonstrate how CPM allows us to perform model comparison to test whether there is stronger evidence for either of the two TD models generating the BOLD data in each of the regions of interest.

In the risk-sensitive decision-making task, there were five stimuli: #1 & #2 resulted in $0 for sure, #3 $0.2 for sure, #4 $0.4 for sure, and #5 was a 50% chance of $0.4 or 0 otherwise. There were two types of trials, forced trials where one of the stimuli was presented to the subject and then realized, and choice trials where they would choose which of two available stimuli was to be realized.

The data provided were average time courses from the left and right nucleus accumbens regions of interest (ROI). Data were provided from 16 subjects with 3 sessions each. We preprocessed the data by regressing out the provided motion parameters, their temporal derivatives, a linear, and a quadratic trend. Before regression, the confounds were z-transformed.

A similar stimulus representation as described above was used, so that TD errors were estimated at the onset of the stimulus and at the time the reward was delivered. For our analysis, we concatenated trial and functional MRI data to generate a continuous time series. We modeled the value for each trial only based on the selected stimulus (i.e., ignoring the not-chosen stimulus in choice trials), so that we could apply the TD-learning models, as if it were a classical conditioning task.

We applied the CPM method using the same risk-sensitive and classical TD learning models to the trial data, ignoring trials where no choice was made. For each region of interest, we performed Bayesian model comparison across participants, comparing the classical TD model against the risk-sensitive model, using the variational Bayesian analysis toolbox. We performed model comparison for the full dataset (all three sessions concatenated). We also compared the data fit of our model to the TD errors of the risk-sensitive and the classical TD model that were provided by Wilson and Niv (2015). To do so, we rounded the individual event onsets to the nearest fMRI volume and created a vector that only had values at the time of an event and 0 otherwise. As values, we used the TD errors of either of the two models. This vector was then convolved with the canonical HRF function provided by SPM to derive a BOLD signal. Note, we did not apply oversampling as typically done in these settings; however, the results should be comparable.

### 5.3 Results

**Fig. 11.**
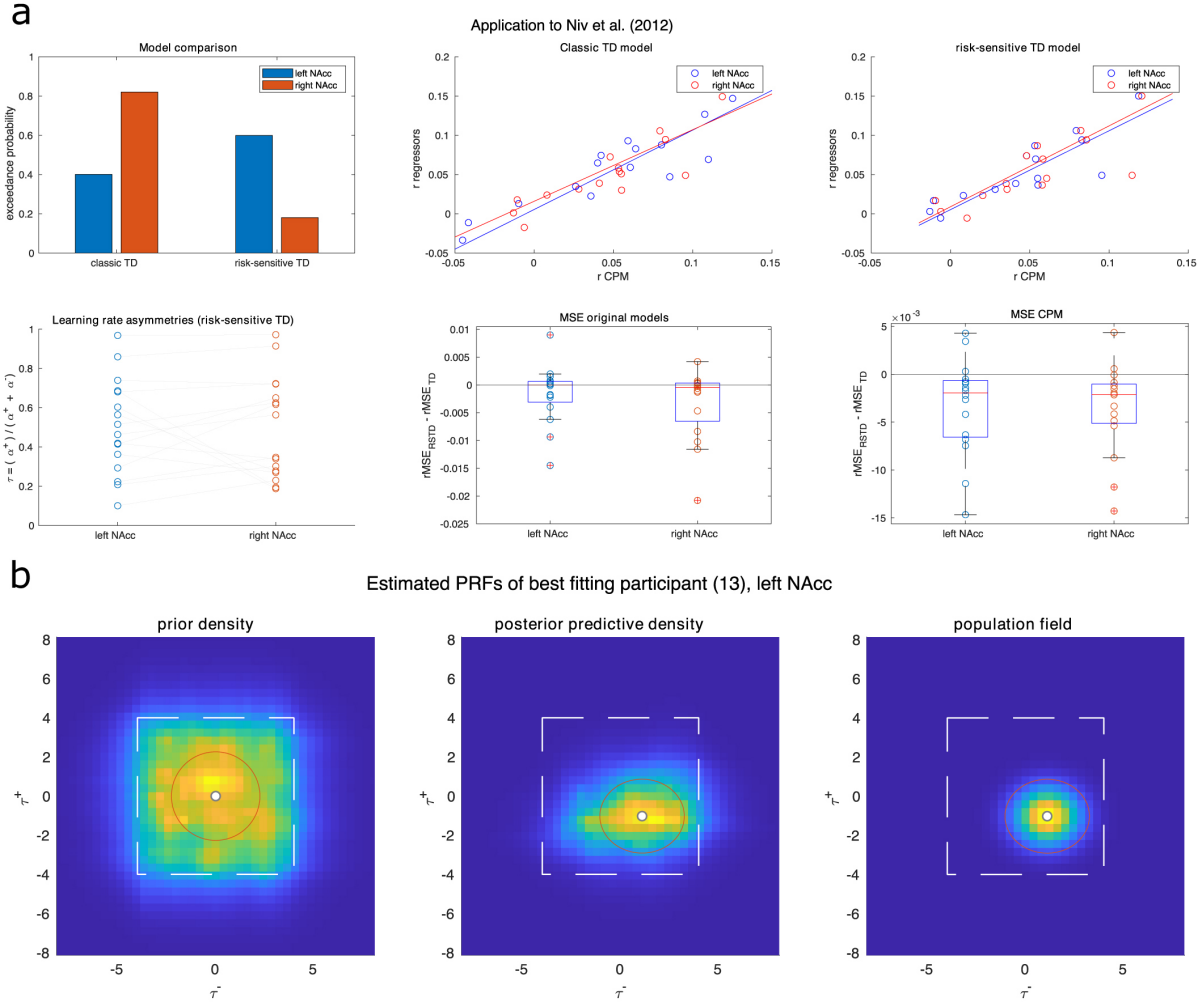
Application to real data from Niv et al. (2012). (a) Upper left shows exceedance probabilities of model comparisons for the left and right NAcc region of interest over 16 participants. The upper middle and right show the correlation of fit statistics (Pearson correlation) of the regressors provided and the CPM model. The lower left panel shows the learning rate asymmetries of the risk-sensitive model in left and right NAcc. Lower middle and right, difference in root mean squared error (of the predicted and actual fMRI signal) between the risk-sensitive and the classical TD model for the provided regressors and CPM estimates, separated by ROI. To allow for comparison between CPM and the other TD-errors, predicted and actual fMRI data were z-scored before calculation of the rMSE. (b) Population fields estimated for the risk-sensitive TD model with the best fit. Left is the prior density of the receptive field (1000 samples), middle is the posterior predictive density (1000 samples), and right displays the population field based on the posterior parameter estimates, which is applied to the data. The dot and the red outline indicate the population field based on the MAP estimates of the location and of spread parameters.

Our proof-of-principle analysis shows that CPM has a comparable model fit to real data, as model-based approaches, that were fitted to behavioral responses (Fig. 11). These results are comparable, even when we use a different model formulation. The correlation between model fits for the classic TD model based on the fitted learning rates and our estimation was high (left NAcc, r=0.8981 , p=0.0000023 ; right NAcc, r=0.906 , p= 0.0000014 ). For the risk-sensitive model, the correlation was still high, but slightly lower (left NAcc, r=0.8714 , p=0.00001 ; right NAcc, r=0.8564 , p=0.00002 ). Our approach, furthermore, allows for different avenues of analyses that have not been possible before. As we estimated the learning rate asymmetry from the BOLD data directly, we can, for example, compare if there is a difference between left and right nucleus accumbens (paired t-test on τ estimates, t(15)=0.189 , p=0.8526 , $BF_{0,1}=5.20585$ ). For each region of interest, we can also calculate which model comes out winning in a model comparison. In our case, there does not seem to be strong evidence in either direction for left NAcc ($BF_{\text{rsTD},\text{classicTD}}=1.495$ ), and substantial evidence in favor of the classic TD model for right NAcc, $BF_{\text{rsTD},\text{classicTD}}=$ 0.2201 ; exceedance probabilities classic TD, left NAcc =0.4008 , right NAcc =0.8196 ; exceedance probabilities risk-sensitive TD, left NAcc =0.5993 , right NAcc =0.1804 ).

### 5.4 Pilotdata

To illustrate how CPM might be applied to real data, we apply our analyses here to an unpublished pilot dataset. Again, these analyses are purely meant for illustration, and we do not make any empirical claims about the results.

#### 5.4.1 Participants

Our study protocol and data collection complied with all relevant ethical regulations stipulated by the capital region of Denmark (reference# H-24015199) under the declaration of Helsinki. Written informed consent was obtained from all participants. We recruited 15 participants as part of a pilot study (see Supplementary Materials C for more details).

#### 5.4.2 Task

We used a similar task as in Niv et al. (2012), following the implementation by Rosenbaum et al. (2022). In this version of the risk-sensitive decision-making task, there were five stimuli: #1 resulted in 0 points for sure, #2 20 for sure, #3 40 for sure, #4 was a 50% chance of 40 or 0 otherwise, and #5 a 50% chance of 80 or 0 otherwise. There were two types of trials, forced trials where one of the stimuli was presented to the participant and had to be selected, and choice trials where the participant would choose which of two available stimuli was to be realized. The points accumulated filled up a bar at the top of the screen, indicating the proportion of 75 DKK the participants could earn during the experiment.

#### 5.4.3 CPM model

We used the same reward prediction error model as described above, modeling the onsets of the stimulus selection feedback and the reward realization as onsets of interest. For this analysis, however, we used a normal prior over β∼N(0, 0.1) , to simplify thresholding for voxel selection. We further initialized the location of the population field using correlation and mean squared error estimates (see Supplementary Materials B).

#### 5.4.4 Results

**Fig. 12.**
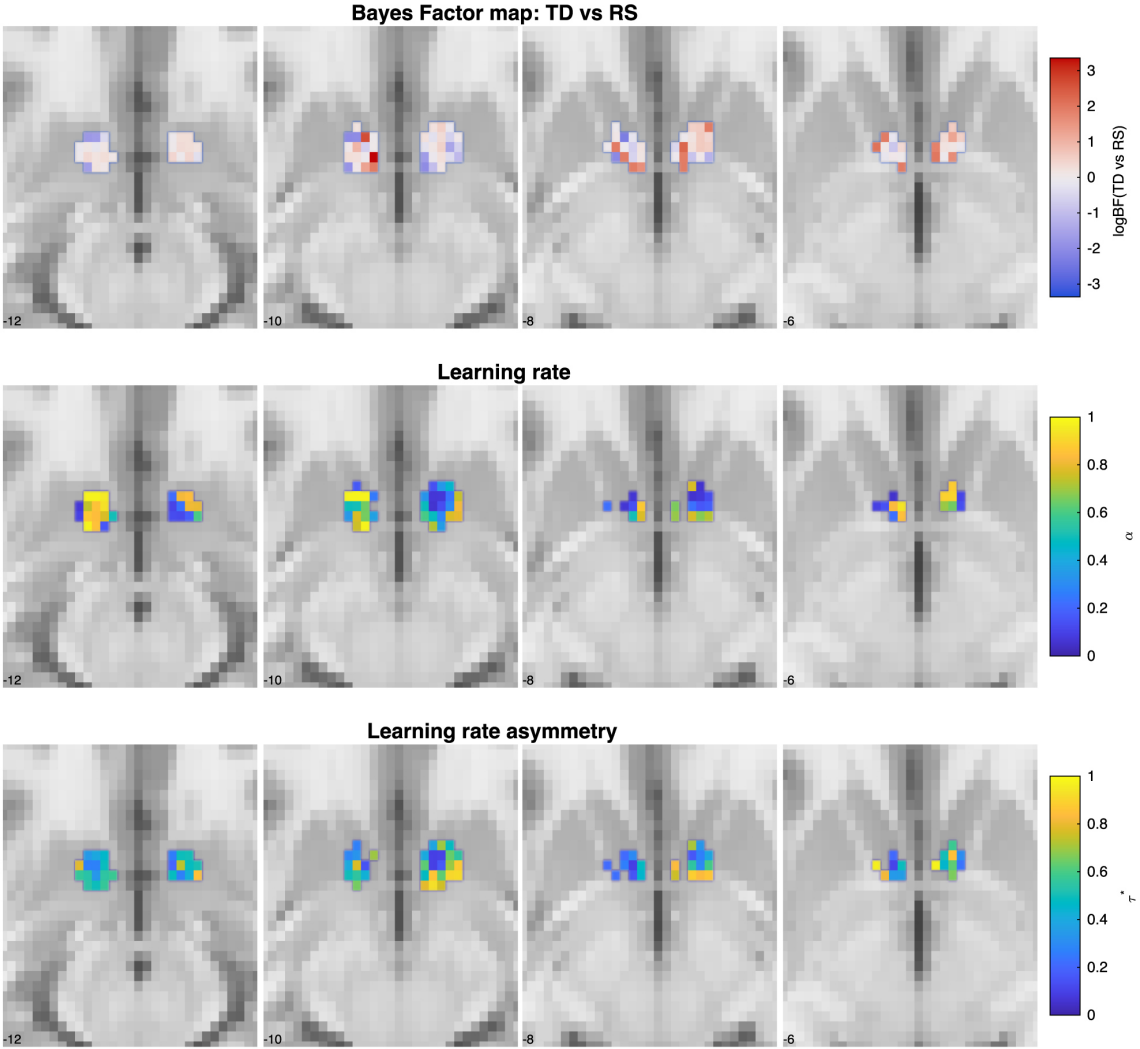
Application to real data from a pilot dataset. The upper row shows the voxelwise log Bayes factor comparing the classical TD model against the risk-sensitive model. Higher values indicate stronger evidence for the classical model. A log Bayes factor of ∼2.3 corresponds to a BF of 10, and ∼3.4 corresponds to a BF of 100. The second row shows the voxelwise posterior point estimates (MAP) for the learning rates of the classical TD model. The third row shows point estimates of the learning rate asymmetry for the risk-sensitive model, calculated as $\tau *=\frac{\alpha^{+}}{\alpha^{+}+\alpha^{-}}$. The higher the learning rate asymmetry score, the higher the learning rate for positive RPE relative to negative RPEs, with a balance between the two at 0.5.

Here, we show the results of the participant with the highest average posterior probability over β for both left and right nucleus accumbens. Voxels were thresholded at a posterior probability for β of p>0.75 (Fig. 12, upper). We show voxelwise estimates of the learning rate (α) (Fig. 12, middle row) for the classic TD model and of the learning rate asymmetry (τ* ) (Fig. 12, lower row). There seems to be generally higher evidence for the classic TD model across voxels in both the left and right nucleus accumbens. Further, there seem to be lower learning rates in the right nucleus accumbens compared to the left and higher learning rates more ventrally compared to higher learning rates in the more dorsal slices. On the other hand, we see more “optimistic” voxels (higher learning rate asymmetry) in the right nucleus accumbens, compared to the left.

## 6 Discussion

In this paper, we have outlined computational parametric mapping as a modeling framework that integrates model-based fMRI and receptive field modeling. Algorithmically, it is only an incremental step to apply the receptive field model to a model with a different domain of application, namely, cognitive rather than sensory models. Nonetheless, the ability to directly estimate cognitive models based on the activity of individual voxels is an important advance. For example, new insights from the reward learning literature indicate that the neural responses following reward are more heterogeneous than previously assumed (Dabney et al., 2020; Meder et al., 2017; Muller et al., 2024). Thus, modeling the neural reward processes as if they were homogeneous channels that are represented by the parameters fit to behavior would be blind to the more detailed neural reality of the underlying processes.

In the development of CPM, we were met with computational challenges when scaling up to the number of voxels that would be useful in fMRI experiments. We found a way to substantially speed up the computations implemented in the SPM12 toolbox. This could be beneficial to users of other Bayesian inference methods implemented in SPM, such as dynamic causal modeling.

Our simulation study shows that we can reliably recover the parameters of cognitive models using CPM. It also demonstrates good model recovery performance for the simulated models tested. Our proof-of-concept analysis of a real dataset reveals that CPM has a similar model fit as a classic model-based fMRI approach, where the model was fitted to behavioral data (Niv et al., 2012). While this is to be expected (Wilson & Niv, 2015), it still is indicative of its construct validity in terms of the latent parameters it can infer. The results of the model comparison here are somewhat ambiguous, finding no winning model for the left nucleus accumbens, and contrary to our assumption, evidence favours the classic TD model for the right nucleus accumbens. As this analysis was done mainly as proof-of-concept, we neither make empirical claims on the topography of results not do we put any emphasis on these results and there are multiple possible reasons why a model with fewer parameters has an advantage over a more complex one (e.g., noise in the signal, parameters that are close to each other, etc.), which we did not investigate here. In a second proof of concept analysis on a pilot dataset, we show that topographic mapping of parameters and voxelwise model comparison is, in principle, possible, but again, we do not want to make empiricial claims about these results at this point.

The simulation and model recovery analyses provide a proof-of-principle for evaluating how models behave under conditions of model mismatch (for an example with a different generative process see Supplementary Materials D). Because the data-generating process is known, the degree of mismatch between generative and fitted models can be explicitly quantified. In the simulated experiments, the ground-truth model consistently exhibits superior predictive adequacy relative to mismatched models. An important exception arises when data are generated by a more complex process that produces outcomes indistinguishable from those generated by a simpler model. For example, when a risk-sensitive model employs identical learning rates for positive and negative prediction errors, the resulting behavior is equivalent to that of the classical single–learning-rate model. In such cases, the simpler model achieves higher model evidence because it captures the same predictions with fewer parameters. This behavior is precisely what one should expect—it reflects the framework functioning correctly under conditions of parameter degeneracy. This nuance underscores that model comparison must always be interpreted in the context of the experimental design and the extent to which models make distinguishable predictions. We advise to make the same efforts in optimizing experimental designs with regards to the latent states and the theoretical BOLD response as one would do for behavioral studies (Wilson & Collins, 2019). There still needs to be more research done on the limitations of our proposed method; we here show that it could be an appropriate tool to not only locate brain regions that are correlated with a specific cognitive process as in classic model-based fMRI (O’Doherty et al., 2007), but also further make inference about the latent parameters that drive these processes, and crucially their anatomical topography. This method now allows us to map parameters of cognitive models to individual voxels in large regions of interest, and to investigate possible topographic motifs and gradients. Our implementation of the toolbox is relatively straightforward, so that previous studies using model-based fMRI could quickly be adapted to be used with CPM—as we did in our application to the data of Niv et al. (2012). The Bayesian framework further allows the voxelwise or regionwise comparison of different computational models, so that hypotheses about the neural implementations of cognitive models in brain processes can be tested directly.

We note that the examples discussed so far deployed Gaussians for modeling population fields; however, the modeling framework can evaluate alternative choices of basis functions, which could be an avenue for exploration. If of scientific interest, the basis function choice can be formulated as a model comparison within the framework. More fundamentally, modeling parameter fields as distributions—rather than estimating single parameter values for each voxel—reflects the biological reality that each voxel contains a population of neurons that may differ in their computational properties. Representing this heterogeneity with a continuous distribution, such as a Gaussian, enables us to make principled inferences about both the central tendency and the uncertainty of the underlying parameter values. This allows us to compute precision-weighted averages across voxels and compare the consistency of parameter estimates within and between regions. Furthermore, estimating a field over parameters permits the construction of population-level hypotheses, such as gradients or motifs in cognitive function, that would be inaccessible with pointwise estimates alone.

Our simulations also reveal another subtlety of the population field structure we deployed: We are using a linearized grid of a latent variable to approximate the native parameter space. The native parameters, however, lead to non-linear changes in the generated data; thus our parameter recovery has some difficulties in recovering the exact locations and larger inaccuracies in recovering the variance of the generative process. However, the main features of interest of the model—the asymmetry of learning rates—can still be recovered accurately. For users, this means that the spread of the field still represents the uncertainty over the parameter space, but it might additionally be skewed by non-linear effects of the parameters of the cognitive model. To test this, we ran an additional simulation study using an example of retinotopic mapping, showing that there is near-perfect recovery of both the location and shape parameters and thus that the method is non-biased if there is a linear effect of the parameters covered by the receptive field (Supplementary Materials A.3, Fig. S3).

It is important to note that, for any cognitive model, the computational complexity of the response space increases exponentially as the dimensionality of the parameter space grows. Specifically, if a grid-based approach is used to discretize a parameter space with d dimensions, where each dimension is sampled at k points, the total number of grid points scales as $O(k^{d})$ . This exponential growth quickly renders exhaustive precomputation infeasible for high-dimensional parameter spaces. The present framework is deliberately specialized for simple cognitive models with a small number of parameters. When fitting to neural data, low-dimensional cognitive models are often the most productive and tractable starting point. For such models, the precomputed grid approach is particularly advantageous because it allows for the efficient reuse of a fixed response space. This leads to computational benefits in terms of both speed and energy efficiency during estimation. However, as the number of parameters increases, the advantage of response space recycling diminishes, as the computational burden of simulating responses over a high-dimensional grid becomes intractable. In such cases, the cost of precomputing the response space scales unfavorably, and alternative estimation methods must be considered. If testing higher-dimensional cognitive models is necessary, these models can instead be estimated via direct fitting, for which we also provide functionality. Notably, in this approach, uncertainty in terms of a spread over the parameter space is no longer explicitly estimated. This trade-off between low-dimensional and high-dimensional cognitive models represents a key challenge for future iterations of the toolbox, requiring further methodological refinements to balance computational efficiency with model complexity.

Similarly to the pRF toolbox, we should rehearse the limitations of variational Bayes here. By using the variational Bayes under the Laplace approximation, we only approximate the probability density distribution over its parameters. The assumption is that the true probability density distribution can be approximated using a multivariate normal distribution, which is different to classical sampling methods (such as MCMC) that can approximate the true probability density distribution over the parameters (Zeidman et al., 2018). However, validations of similar methods using sampling have been conducted, showing that the Laplace approximation is generally appropriate (Friston et al., 2007).

The paper here focuses on fMRI, but there is nothing inherent about the suitability of the approach to this one data modality. The applicability to fMRI data is achieved via the observation model (here, the balloon model). To apply to other data modalities, the observation model would need to be adapted. There are possible extensions to other data modalities such as EEG, MEG, or even calcium imaging techniques. The method could also extend to datasets in which multiple data modalities are collected simultaneously, where the neuronal model is taken as a common latent cause of both data modality streams.

We, thus, encourage and support the development of this class of computational parametric model beyond its colloquial application to fMRI data.

## Supplementary Material

Supplementary Material

## Data Availability

All code for this study is available on Github https://github.com/ergEx/BayespRF_CPM. The code for the simulations can be found in the SIMULATIONS folder. Data for Niv et al. (2012) / Wilson and Niv (2015) are available at https://journals.plos.org/ploscompbiol/article?id=10.1371/journal.pcbi.1004237. The data for the retinotopic simulation were taken from the SAMSRF ((Schwarzkopf, 2016) example data, available at https://zenodo.org/records/163582, and use the apsBars.mat file in the pRF folder. Raw and preprocessed data for the topographic maps are available on request from O.J.H. The data are not publicly available at this point in time due to data protection reasons.
