# Supplementary material for: Computational parametric mapping of functional neuroimaging data
Source: Imaging Neurosci (Camb). 2026 Mar 4;4:IMAG.a.1130. doi: 10.1162/IMAG.a.1130 (PMC12961307; doi:10.1162/IMAG.a.1130)
Supplement: Supplementary Material [file IMAG.a.1130_supp.pdf]

Supplementary materials: Computational parametric mapping of functional neuroimaging data

Simon R. Steinkamp,[1*] Iyadh Chaker,[2] Felix Hubert,[3] David Meder,[1] Oliver J. Hulme,[1,4,5]

[1] Danish Research Centre for Magnetic Resonance,

Department of Radiology and Nuclear Medicine,

Copenhagen University Hospital Amager and Hvidovre, Copenhagen, Denmark.

[2] Department of Physics, University of Trento, Trento, Italy.

[3] Department of Basic Neuroscience, University of Geneva, Geneva, Switzerland.

[4] London Mathematical Laboratory, London, UK.

[5] Department of Psychology, University of Copenhagen, Copenhagen, Denmark.

*Corresponding author: simons@drcmr.dk

February 16, 2026

Supplementary materials

A Simulations and optimisation

A.1 Posterior correlation of TD models

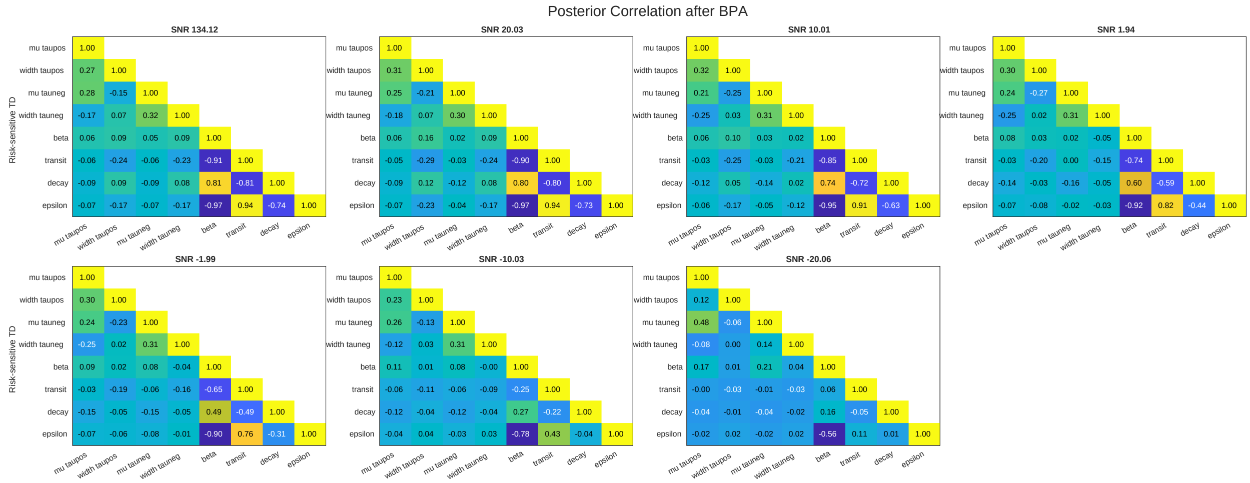

Fig. S1: Posterior parameter correlation of and risk-sensitive TD (lower row) models, estimated by calculating Bayesian Parameter Average and transforming the posterior covariance matrix into a correlation matrix. Each column represents one of the noise levels. Note that we use the latent parameters here (indicated by names like "lmu_tau"). Here we removed recovered models, where τ^+ and τ^- were the same.

A.2 Defining the population field

The latent transforms and requirements for the population field need to be taken into account before setting up the discrete parameter space P and the population field. In this context, we can define a possible and a probable parameter space. Where the former spans all parameters in P, and the latter imposes limits on the parameter extent (i.e., only the parameters we deem probable). The values of the computational model $\mathcal{S}$ over the experimental session are precomputed on a discretized subset of the model parameter space P, which requires finite support $[p_{min}, p_{max}]$ and either the resolution (n) or the step size r, which defines a discrete subset of values for each parameter p (where $r = (p_{max} - p_{min})/n$). These hyperparameters have to be decided on by the user, taking the desired precision and computational costs into account for choosing the resolution and finding reasonable boundaries. We will call this parameter space the *possible parameter space*, as these are all the values that in principle, could be

covered by the population field. Additionally, we might want to define a *probable parameter space*, this space limits the location of the population field and is defined by μ_{min} and μ_{max}. The choice of these hyperparameters directly affects the population field. In our model, we define the minimum standard deviation (σ_{min}) of the population field as $\sigma_{min} = r/2$. This means that the smallest (i.e. most precise) population field covers the adjacent points around its location with 95 % density. Further, we want to define σ_{max} so that the largest extent of the population field covers at least 95 % of the parameter space over μ. We thus define $\sigma_{max} \geq (\mu_{max} - \mu_{min})/4$. Choosing σ_{max} this way has further consequences. Ideally, we have defined p_{min} and p_{max}, so that the precomputed grid is significantly larger than μ_{min} and μ_{max}. This means that the parameter grid of the possible parameter space (P) is larger than the probable parameter space (μ). In the case that $p_{max} - p_{min} \geq (\mu_{max} - \mu_{min}) * 2$, we can safely assume an upper limit for σ_{max} as the half-distance between the probable and the possible parameter space (under the assumption of symmetry around the centre for μ and p), we can set $\sigma_{max} = (p_{max} - \mu_{max})/2$. As can be seen in Fig. S2, this means that the population field $N(\mu_{max}, \sigma_{max})$ can be placed at the edges of the probable parameter space μ and is not significantly restricted by the borders of the possible parameter space p. In other cases where $p_{max} - p_{min} \leq (\mu_{max} - \mu_{min}) * 2$, we define $\sigma_{max} = (\mu_{max} - \mu_{min})/4$, under the caveat that the population field $N(\mu_{max}, \sigma_{max})$, is restricted by the borders of P. Note that $N(\mu_{max}, \sigma_{max})$ will still be a probability mass function over P, as we normalize the population field to sum to 1; however, it loses some interpretability as it might not constitute a Gaussian anymore, as the extent of the population field is compressed by the borders of the population field.

In general, we set the prior value of the population field's spread to $\sigma_{prior} = (\sigma_{max} - \sigma_{min})$, which in practice means setting the latent parameter $l\sigma = 0$, i.e., so that the prior value of σ is at the centre of the latent Gaussian prior. $\sigma_{\text{prior}} = (\mu_{max} - \mu_{min})/2$. If the probable parameter space μ is not defined, we set $\mu_{min} = p_{min}$ and $\mu_{max} = p_{max}$.

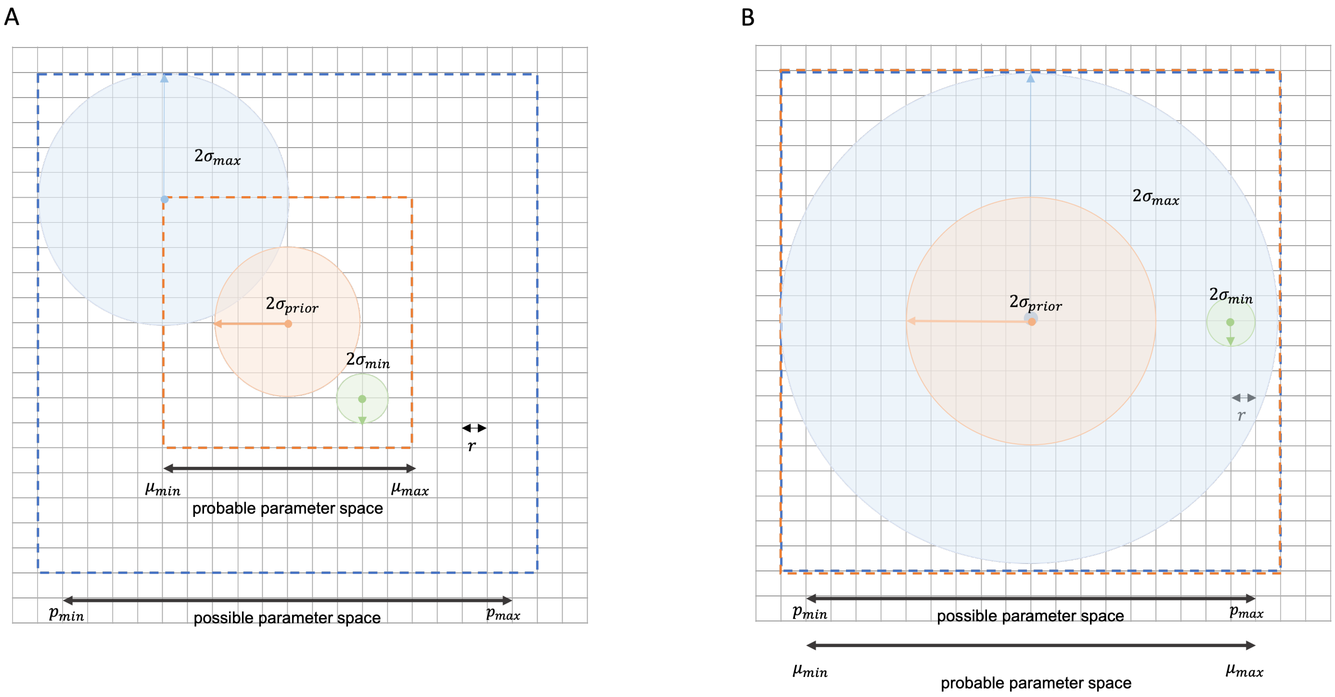

Fig. S2: **Schematic of the parameter space and the possible extensions of the population field.** a) shows a case where the probable and possible parameter spaces are well-defined, and where the extent of the population field is always a Gaussian probability mass function. b) shows a case where probable and possible parameter spaces are the same. This allows the population field to cover the whole grid. This, however, sacrifices the Gaussian interpretability of the population field.

A.3 Simulating Retinotopy Data

We also tested our CPM approach on an example from retinotopic mapping, the SAMSRF (Schwarzkopf, 2016) example retinotopy data. The approach differs slightly from the actual CPM approach described in the paper. Since this example uses actual images to estimate the coordinates and spreads of the population receptive field, and these images are given for each fMRI scan, the given image stream of intensities is equivalent to our precomputed input S. The example data provides the images for each time-point corresponding to their functional data; however, we added the following preprocessing steps to make them fit with the more general framework of CPM. The provided images were down-sampled to 15 by 15 pixels, and the resulting image was padded with 8 zero pixels on all sides. This was to ensure that most of the image lies in the probable parameters space (x and y coordinates between -10 and 10).

Again, we simulated 16 voxels at different locations and with different spreads at the noise levels described in the methods sections. Again, CPM recovers the location parameters well. Compared to the reward learning examples, however, the shape of the population field model used in the generative process is recovered with higher fidelity. This demonstrates that the use of non-linear parameter transforms after using the grid may lead to some issues in recovering the generative process.

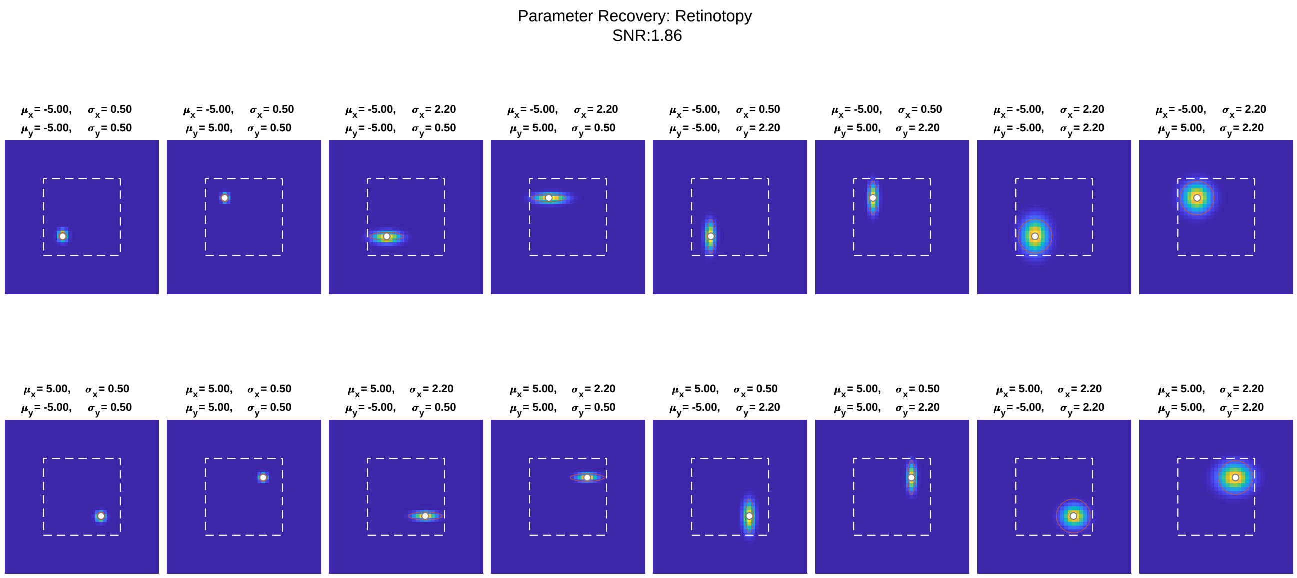

Fig. S3: Parameter recovery of a simulated retinotopy problem at an SNR close to 2. We here see the near-perfect recovery of both the spread of the simulated population field as well as the location.

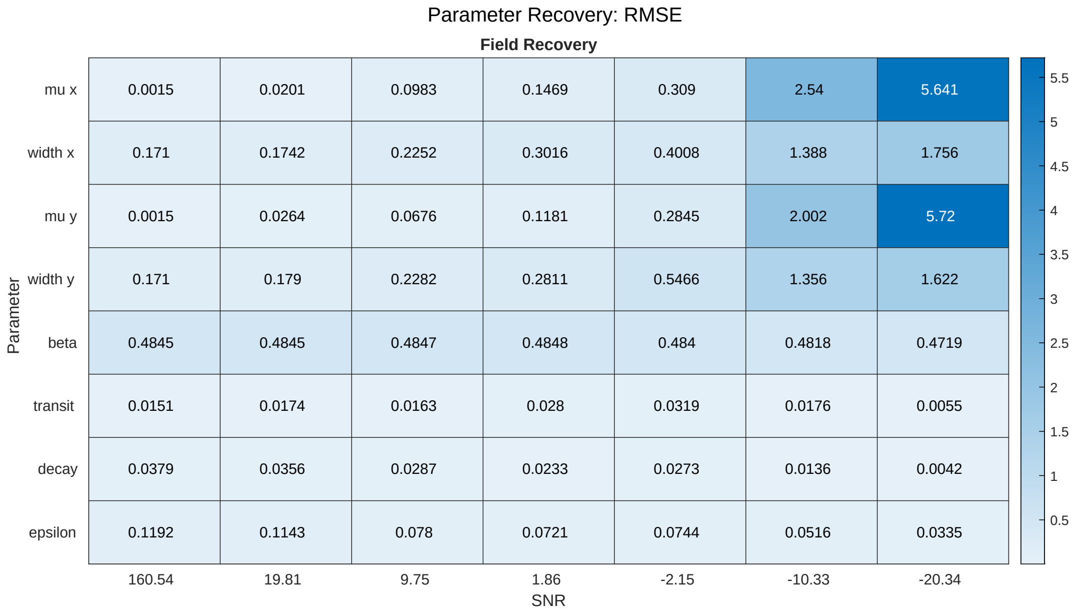

Fig. S4: Root mean squared error of the parameter recovery for the retinotopic simulation.

B Initialization via GLM

We also support a functionality of the BayesPRF toolbox (Zeidman et al., 2018) to initialize the inference procedure, using a first guess of the location parameter estimates. This means we create a mesh grid of $x = [-2, 2]$ with a step size of 0.2 of the population response's location in latent space. For each parameter combination, we create the population field at that location (using the prior width) and simulate the neural response (assuming $\beta = 1$). The neural response is then convolved with the canonical HRF implemented in SPM. We then run a regression model (BOLD signal + intercept) against the actual BOLD signal at that voxel / region. We then select the parameter combination that minizmises the mean squared error of the regression's residuals, these parameters are then used as a first guess for the inference.

C Pilot data

C.1 Data

The task-related functional images were acquired with a multi-echo planar imaging sequence (TR = 1800 ms; TE1 = 14 ms; TE2 = 31.75 ms, TE3 = 49.5 ms; flip angle = 78°; matrix size 78 × 78; Field of

view = 210 mm; 44 axial slices; interleaved slice acquisition; slice thickness = 2.67 mm with 10% gap, in-plane resolution = 2.67 mm; bandwidth = 2465 Hz/pixel; iPAT factor 2; multi-band factor 2; 690 volumes). Data were aligned to a T1w image derived from an MP2RAGE sequence.

C.1.1 Preprocesing

Results included in this manuscript come from preprocessing performed using *fMRIPrep* 25.0.0 (Esteban et al. (2019); Esteban et al. (2018); RRID:SCR_016216), which is based on *Nipype* 1.9.2 (K. Gorgolewski et al. (2011); K. J. Gorgolewski et al. (2018); RRID:SCR_002502).

C.1.2 Copyright Waiver

The boilerplate text below was automatically generated by fMRIPrep with the express intention that users should copy and paste this text into their manuscripts *unchanged*. It is released under the CC0 license.

Preprocessing of B0 inhomogeneity mappings A total of 8 fieldmaps were found available within the input BIDS structure for this particular subject. A *B0*-nonuniformity map (or *fieldmap*) was estimated based on two (or more) echo-planar imaging (EPI) references with `topup` (Andersson et al. (2003); FSL None).

Anatomical data preprocessing A total of 1 T1-weighted (T1w) images were found within the input BIDS dataset. The T1w image was corrected for intensity non-uniformity (INU) with `N4BiasFieldCorrection` (Tustison et al., 2010), distributed with ANTs 2.5.4 (Avants et al., 2008, RRID:SCR_004757), and used as T1w-reference throughout the workflow. The T1w-reference was then skull-stripped with a *Nipype* implementation of the `antsBrainExtraction.sh` workflow (from ANTs), using OASIS30ANTs as target template. Brain tissue segmentation of cerebrospinal fluid (CSF), white-matter (WM) and gray-matter (GM) was performed on the brain-extracted T1w using `fast` (FSL (version unknown), RRID:SCR_002823, Zhang et al., 2001). Volume-based spatial normalization to one standard space (MNI152NLin2009cAsym) was performed through nonlinear registration with `antsRegistration` (ANTs 2.5.4), using brain-extracted versions of both T1w reference and the T1w template. The following template was were selected for spatial normalization and accessed with *TemplateFlow* (24.2.2, Ciric et al., 2022): *ICBM 152 Nonlinear Asymmetrical template version 2009c* [Fonov et al. (2009), RRID:SCR_008796; TemplateFlow ID: MNI152NLin2009cAsym].

Functional data preprocessing For each of the 8 BOLD runs found per subject (across all tasks and sessions), the following preprocessing was performed. First, a reference volume was generated from the shortest echo of the BOLD run, using a custom methodology of *fMRIPrep*, for use in head motion correction. Head-motion parameters with respect to the BOLD reference (transformation matrices, and six corresponding rotation and translation parameters) are estimated before any spatiotemporal filtering using `mcflirt` (FSL , Jenkinson et al., 2002). The estimated *fieldmap* was then aligned with rigid-registration to the target EPI (echo-planar imaging) reference run. The field coefficients were mapped on to the reference EPI using the transform. The BOLD reference was then co-registered to the T1w reference using `mri_coreg` (FreeSurfer) followed by `flirt` (FSL , Jenkinson & Smith, 2001) with the boundary-based registration (Greve & Fischl, 2009) cost-function. Co-registration was configured with six degrees of freedom. Several confounding time-series were calculated based on the *preprocessed BOLD*: framewise displacement (FD), DVARS and three region-wise global signals. FD was computed using two formulations following Power (absolute sum of relative motions, Power et al. (2014)) and Jenkinson (relative root mean square displacement between affines, Jenkinson et al. (2002)). FD and DVARS are calculated for each functional run, both using their implementations in *Nipype* (following the definitions by Power et al., 2014). The three global signals are extracted within the CSF, the WM, and the whole-brain masks. Additionally, a set of physiological regressors were extracted to allow for component-based noise correction (*CompCor*, Behzadi et al., 2007). Principal components are estimated after high-pass filtering the *preprocessed BOLD* time-series (using a discrete cosine filter with 128s cut-off) for the two *CompCor* variants: temporal (tCompCor) and anatomical (aCompCor). tCompCor components are then calculated from the top 2% variable voxels within the brain mask. For aCompCor, three probabilistic masks (CSF, WM and combined CSF+WM) are generated in anatomical space. The implementation differs from that of Behzadi et al. in that instead of eroding the masks by 2 pixels on BOLD space, a mask of pixels that likely contain a volume fraction of GM is subtracted from the aCompCor masks. This mask is obtained by thresholding the corresponding partial volume map at 0.05, and it ensures components are not extracted from voxels containing a minimal fraction of GM. Finally, these masks are resampled into BOLD space and binarized by thresholding at 0.99 (as in the original implementation). Components are also calculated separately within the WM and CSF masks. For each CompCor decomposition, the k components with the largest singular values are retained, such that the retained components' time series are sufficient to explain 50 percent of variance across the nuisance mask (CSF, WM, combined, or temporal). The remaining components are dropped from consideration. The head-motion estimates calculated in the correction step were also placed within the corresponding confounds file. The confound time series derived from head motion estimates and global signals were expanded with the inclusion

of temporal derivatives and quadratic terms for each (Satterthwaite et al., 2013). Frames that exceeded a threshold of 0.5 mm FD or 1.5 standardized DVARS were annotated as motion outliers. Additional nuisance timeseries are calculated by means of principal components analysis of the signal found within a thin band (*crown*) of voxels around the edge of the brain, as proposed by (Patriat et al., 2017). All resamplings can be performed with *a single interpolation step* by composing all the pertinent transformations (i.e. head-motion transform matrices, susceptibility distortion correction when available, and co-registrations to anatomical and output spaces). Gridded (volumetric) resamplings were performed using `nitransforms`, configured with cubic B-spline interpolation.

Many internal operations of *fMRIPrep* use *Nilearn* 0.11.1 (Abraham et al., 2014, RRID:SCR_001362), mostly within the functional processing workflow. For more details of the pipeline, see the section corresponding to workflows in *fMRIPrep*'s documentation.

C.2 Preparation

The preprocessed, optimally combined images in MNI Space (2mm resolution) were submitted to a first-level analysis in SPM12, where the design matrix contained the six rotation and realignment parameters and framewise displacement (all derived from fmriprep). We used standard SPM parameters, but explicitly set the number of oversampling time points to the number of slices (44) and the reference time-point to the middle slice (t0 $= 44/2$). After model estimation, we extracted the voxelwise residual timeseries using a binary mask of the left and right nucleus accumbens. The masks were generated from the Pauli atlas (Pauli et al., 2018), using *nilearn*(Abraham et al., 2014, RRID:SCR_001362).

C.3 Visualization

Topographic maps were created using a customized version of https://github.com/dmascali/BrainSlicer, which can be found at https://github.com/SRSteinkamp/BrainSlicer/tree/zoom_pdf. The customization pertains to zooming into the plotted slices and allowing the export to pdf.

D Model recovery

The model recovery in the main text already covers the models of interest and their degenerate aspects (that is models where the risk-sensitive model mimics the temporal-difference learning model. Another

question arises, if the recovery process can differentiate generative processes (Wilson & Collins, 2019). While Rescorla-Wagner learning rules and temporal difference learning, which track the average reward rate are the main models used to investigate Pavlovian learning process in the brain, which are the main focus of our simulation (Niv, 2009; O'Doherty et al., 2003; Schultz, 2024), alternative models, such as decay learning, that track cumulative rewards have not been investigated as much (Don et al., 2022; Worthy et al., 2018).

In this additional analyses, we show that computational parametric mapping can, at least theoretically, differentiate a decay model from the delta learning rule models that we already discussed in the main text. Our implementation of the decay model, is based on the paper by Worthy et al., 2018:

$$V_{t+1}^{c} = \begin{cases} \eta * V_t^c + r_t, & \text{if } c = s \\ \eta * V_t^c & \text{otherwise,} \end{cases}$$

Where s is the stimulus shown at time t and c is the stimulus identity and $\eta \in [0, 1]$ is the decay rate. To keep the amount of information in the BOLD signal similar to our TD models, we simulate pseudo reward prediction errors as:

$$\delta_t = r_t - V_t^s.$$

We modeled V_t at the onset of the stimulus and δ_t at reward onset. For this simulation, we used the same settings (parameters, noise, parameter transformations, and boundaries) as for the classical TD model in the simulation study, thus modeling proceses, that have a decay rate η of approximately 0.25 and 0.75 ($\mu_\xi \in \{-1.0986, 1.0986\}$, with different widths $\sigma_\xi \in \{0.4394, 0.7324\}$, where ξ is a transformation of η, akin to the transformation of α to τ in the main simulation.

Adding this new model to the simulation procedure described in the main text, we show here the results of the Bayesian model comparison and the results of the parameter recovery of the decay model.

Our simulations show, that our method can differentiate the two generative processes (TD learning vs decay rule) and we can recover parameters of the decay model with a comparable accuracy as for parameters in the main study. The setting of our simulation study, however, is not ideal to test differences between these two learning process, as we deal here with a Pavlovian conditioning experiment, without stimulus selection and relatively sparse stimulus presentations (nine different images across 168 trials), thus the model creates distinct signal trajectories compared to the TD learning models. Although we are able to differentiate between different generative processes in principle, in practice the method's ability to do so is dependent on the the task and experimental implementation in question. Model and parameter

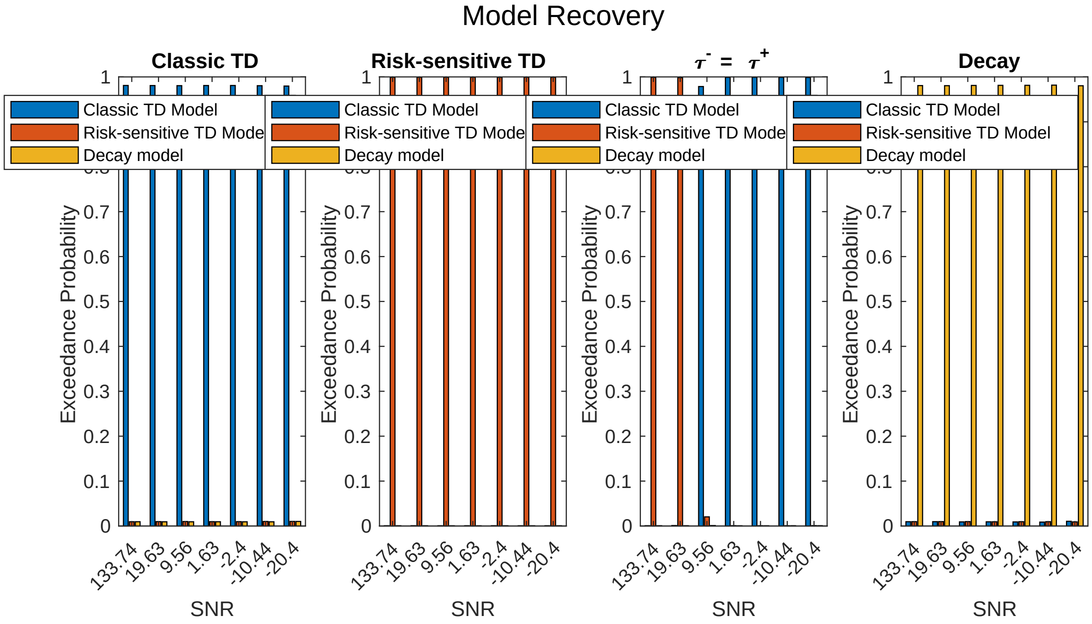

Fig. S5: Exceedance probabilities of the simulation study, including a decay model, representing a different generative process. The results indicate a near perfect separation of the decay model from the other generative processes.

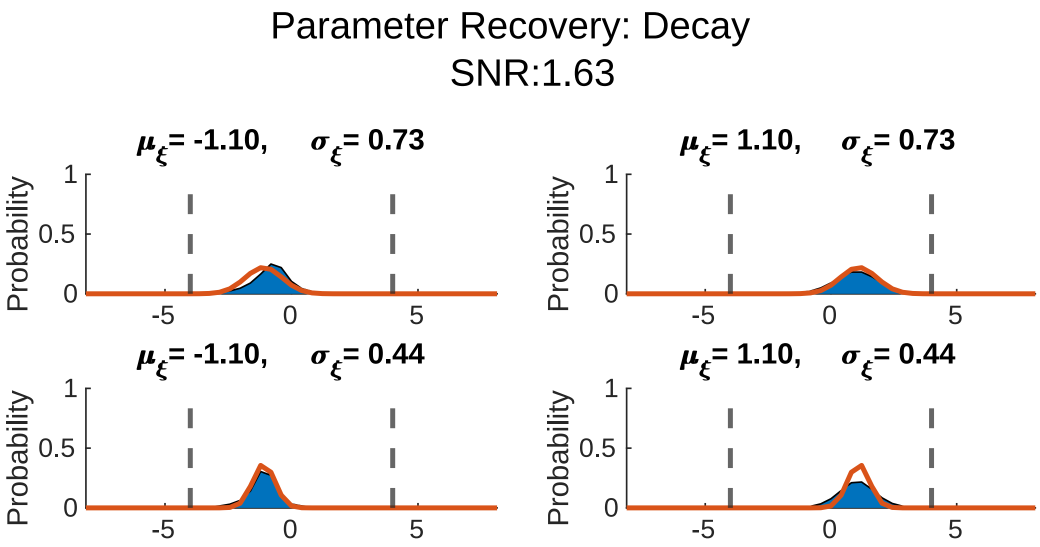

Fig. S6: Parameter recovery of the decay model. As in the main text, the distribution in blue is the recovered population field by our method and orange describes the generative process.

recovery are therefore crucial steps that should be performed for each new experimental design and ideally before data collection (Wilson & Collins, 2019).

E Optimization of computational overhead

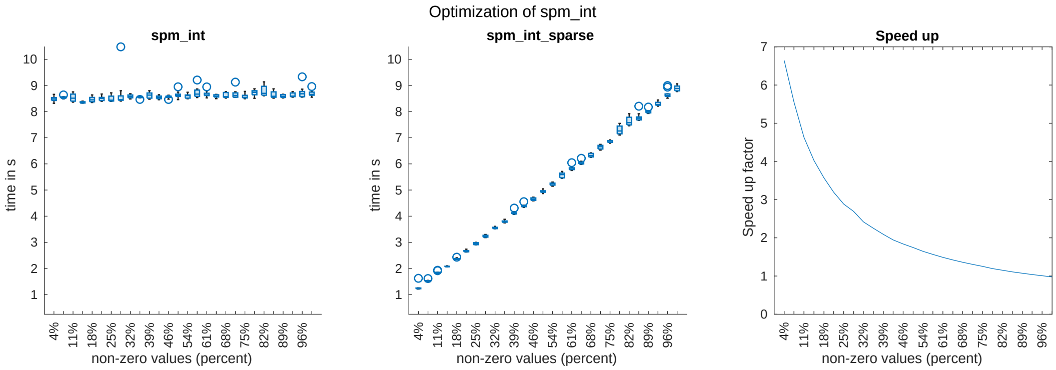

Fig. S7: Speed up gained against the proportion of non-zero values. For 4% non-zero values, the modified spm_int is multiple times faster than normal execution. This graph is specific to the extended hemodynamic Balloon model. The speed-up will be lower for more complex models (larger state vectors). However, the inverse relationship between sparsity and speed-up holds.

Below we present the code of the optimized integration function. The speed-up is proportional to the sparsity of the signal that needs to be integrated. The function can also be found on GitHub https://github.com/ergEx/BayespRF_CPM/blob/master/toolbox/cpm/spm_int_sparse.m

```
function [y] = spm_int_sparse(P, M, U)
    % integrates a MIMO bilinear system dx/dt = f(x,u) = A*x + B*x*u + Cu + D;
    % FORMAT [y] = spm_int(P,M,U)
    % P   - model parameters
    % M   - model structure
    %   M.delays - sampling delays (s); a vector with a delay for each output
    %
    % U   - input structure or matrix
    %
    % y   - response y = g(x,u,P)
    % ------------------------------------------------------------------------
    % Integrates the bilinear approximation to the MIMO system described by
    %
    %     dx/dt = f(x,u,P) = A*x + u*B*x + C*u + D
    %     y     = g(x,u,P) = L*x;
    %
    % at v = M.ns is the number of samples [default v = size(U.u,1)]
    %
    % spm_int will also handle static observation models by evaluating
    % g(x,u,P).  It will also handle timing delays if specified in M.delays
    %
```

```
% ————————————————————————————————————————————————————————————————————————————
%
% SPM solvers or integrators
%
% spm_int_ode:  uses ode45 (or ode113) which are one and multi-step solvers
% respectively.  They can be used for any ODEs, where the Jacobian is
% unknown or difficult to compute; however, they may be slow.
%
% spm_int_J: uses an explicit Jacobian-based update scheme that preserves
% nonlinearities in the ODE: dx = (expm(dt*J) - I)*inv(J)*f.  If the
% equations of motion return J = df/dx, it will be used; otherwise it is
% evaluated numerically, using spm_diff at each time point.  This scheme is
% infallible but potentially slow, if the Jacobian is not available (calls
% spm_dx).
%
% spm_int_E: As for spm_int_J but uses the eigensystem of J(x(0)) to eschew
% matrix exponentials and inversion during the integration. It is probably
% the best compromise, if the Jacobian is not available explicitly.
%
% spm_int_B: As for spm_int_J but uses a first-order approximation to J
% based on J(x(t)) = J(x(0)) + dJdx*x(t).
%
% spm_int_L: As for spm_int_B but uses J(x(0)).
%
% spm_int_U: like spm_int_J but only evaluates J when the input changes.
% This can be useful if input changes are sparse (e.g., boxcar functions).
% It is used primarily for integrating EEG models
%
% spm_int: Fast integrator that uses a bilinear approximation to the
% Jacobian evaluated using spm_bireduce. This routine will also allow for
% sparse sampling of the solution and delays in observing outputs. It is
% used primarily for integrating fMRI models (see also spm_int_D)
% ---------------------------------------------------------------------------
% Copyright (C) 2008 Wellcome Trust Centre for Neuroimaging

% Karl Friston
% $Id: spm_int.m 6856 2016-08-10 17:55:05Z karl $
%
% Optimized by: Iyadh Chaker, 2022

% convert U to U.u if necessary
% ————————————————————————————————————————————————————————————————————————————
```

```
if ~isstruct(U)
    u.u = U;
    U = u;
end
try
    dt = U.dt;
catch
    U.dt = 1;
end

% number of times to sample (v) and number of microtime bins (u)
% ---------------------------------------------------------------
u        = size(U.u, 1);
try
    v = M.ns;
catch
    v = u;
end

% get expansion point
% ---------------------------------------------------------------
x = [1; spm_vec(M.x)];

% add [0] states if not specified
% ---------------------------------------------------------------
try
    M.f = spm_funcheck(M.f);
catch
    M.f = @(x, u, P, M) sparse(0, 1);
    M.x = sparse(0, 0);
end

% output nonlinearity, if specified
% ---------------------------------------------------------------
try
    g   = spm_funcheck(M.g);
catch
    g   = @(x, u, P, M) x;
    M.g = g;
end

% Bilinear approximation (1st order)
```

```
% ----------------------------------------------------------------

[M0, M1] = spm_bireduce(M, P);

m         = length(M1);                    % m inputs

% delays
% ----------------------------------------------------------------
try
    D  = max(round(M.delays / U.dt), 1);
catch
    D  = ones(M.l, 1) * round(u / v);
end

% Evaluation times (t) and indicator array for inputs (su) and output (sy)
% ================================================================

% get times that the input changes
% ----------------------------------------------------------------
i      = [1 (1 + find(any(diff(U.u), 2))')];
su     = sparse(1, i, 1, 1, u);

% get times that the response is sampled
% ----------------------------------------------------------------
s      = ceil((0:v - 1) * u / v);
for j = 1:M.l
    i         = s + D(j);
    sy(j, :) = sparse(1, i, 1:v, 1, u);
end

% time in seconds
% ----------------------------------------------------------------
t      = find(su | any(sy));
su     = full(su(:, t));
sy     = full(sy(:, t));
dt     = [diff(t) 0] * U.dt;

% Integrate
% ----------------------------------------------------------------
y      = zeros(M.l, v);
J      = M0;
U.u    = full(U.u);
```

```
J_old = 0;
E0 = spm_expm(J * dt(1));

for i = 1:length(t)

    % input dependent changes in Jacobian
    % --------------------------------------------------------------------------
    if su(:, i)
        u     = U.u(t(i), :);
        J     = M0;
        for j = 1:m
            J = J + u(j) * M1{j};
        end
    end

    % output sampled
    % --------------------------------------------------------------------------
    if any(sy(:, i))
        q      = spm_unvec(x(2:end), M.x);
        q      = spm_vec(g(q, u, P, M));
        j      = find(sy(:, i));
        s      = sy(j(1), i);
        y(j, s) = q(j);
    end

    if ~(isequal(J_old, J) & dt(i))
        if J == M0
            E = E0;
        else
            E = spm_expm(J * dt(i));
        end

        J_old = J;
    end

    x = E * x;
    % compute updated states x = expm(J*dt)*x;
    % --------------------------------------------------------------------------

    % check for convergence
    % --------------------------------------------------------------------------
    if norm(x, 1) > 1e6
```

```
            break
        end

    end
    y       = real(y');
```

References

Abraham, A., Pedregosa, F., Eickenberg, M., Gervais, P., Mueller, A., Kossaifi, J., Gramfort, A., Thirion, B., & Varoquaux, G. (2014). Machine learning for neuroimaging with scikit-learn. *Frontiers in Neuroinformatics*, *8*. https://doi.org/10.3389/fninf.2014.00014

Andersson, J. L., Skare, S., & Ashburner, J. (2003). How to correct susceptibility distortions in spin-echo echo-planar images: Application to diffusion tensor imaging. *NeuroImage*, *20*(2), 870–888. https://doi.org/10.1016/S1053-8119(03)00336-7

Avants, B., Epstein, C., Grossman, M., & Gee, J. (2008). Symmetric diffeomorphic image registration with cross-correlation: Evaluating automated labeling of elderly and neurodegenerative brain. *Medical Image Analysis*, *12*(1), 26–41. https://doi.org/10.1016/j.media.2007.06.004

Behzadi, Y., Restom, K., Liau, J., & Liu, T. T. (2007). A component based noise correction method (CompCor) for BOLD and perfusion based fmri. *NeuroImage*, *37*(1), 90–101. https://doi.org/10.1016/j.neuroimage.2007.04.042

Ciric, R., Thompson, W. H., Lorenz, R., Goncalves, M., MacNicol, E., Markiewicz, C. J., Halchenko, Y. O., Ghosh, S. S., Gorgolewski, K. J., Poldrack, R. A., & Esteban, O. (2022). TemplateFlow: FAIR-sharing of multi-scale, multi-species brain models. *Nature Methods*, *19*, 1568–1571. https://doi.org/10.1038/s41592-022-01681-2

Don, H. J., Davis, T., Ray, K. L., McMahon, M. C., Cornwall, A. C., Schnyer, D. M., & Worthy, D. A. (2022). Neural regions associated with gain-loss frequency and average reward in older and younger adults. *Neurobiology of Aging*, *109*, 247–258. https://doi.org/10.1016/j.neurobiolaging.2021.10.001

Esteban, O., Blair, R., Markiewicz, C. J., Berleant, S. L., Moodie, C., Ma, F., Isik, A. I., Erramuzpe, A., Kent, M., James D. andGoncalves, DuPre, E., Sitek, K. R., Gomez, D. E. P., Lurie, D. J., Ye, Z., Poldrack, R. A., & Gorgolewski, K. J. (2018). Fmriprep. *Software*. https://doi.org/10.5281/zenodo.852659

Esteban, O., Markiewicz, C., Blair, R. W., Moodie, C., Isik, A. I., Erramuzpe Aliaga, A., Kent, J., Goncalves, M., DuPre, E., Snyder, M., Oya, H., Ghosh, S., Wright, J., Durnez, J., Poldrack,

R., & Gorgolewski, K. J. (2019). fMRIPrep: A robust preprocessing pipeline for functional MRI. *Nature Methods*, *16*, 111–116. https://doi.org/10.1038/s41592-018-0235-4

Fonov, V., Evans, A., McKinstry, R., Almli, C., & Collins, D. (2009). Unbiased nonlinear average age-appropriate brain templates from birth to adulthood. *NeuroImage*, *47, Supplement 1*, S102. https://doi.org/10.1016/S1053-8119(09)70884-5

Gorgolewski, K., Burns, C. D., Madison, C., Clark, D., Halchenko, Y. O., Waskom, M. L., & Ghosh, S. (2011). Nipype: A flexible, lightweight and extensible neuroimaging data processing framework in python. *Frontiers in Neuroinformatics*, *5*, 13. https://doi.org/10.3389/fninf.2011.00013

Gorgolewski, K. J., Esteban, O., Markiewicz, C. J., Ziegler, E., Ellis, D. G., Notter, M. P., Jarecka, D., Johnson, H., Burns, C., Manhães-Savio, A., Hamalainen, C., Yvernault, B., Salo, T., Jordan, K., Goncalves, M., Waskom, M., Clark, D., Wong, J., Loney, F., . . . Ghosh, S. (2018). Nipype. *Software*. https://doi.org/10.5281/zenodo.596855

Greve, D. N., & Fischl, B. (2009). Accurate and robust brain image alignment using boundary-based registration. *NeuroImage*, *48*(1), 63–72. https://doi.org/10.1016/j.neuroimage.2009.06.060

Jenkinson, M., Bannister, P., Brady, M., & Smith, S. (2002). Improved optimization for the robust and accurate linear registration and motion correction of brain images. *NeuroImage*, *17*(2), 825–841. https://doi.org/10.1006/nimg.2002.1132

Jenkinson, M., & Smith, S. (2001). A global optimisation method for robust affine registration of brain images. *Medical Image Analysis*, *5*(2), 143–156. https://doi.org/10.1016/S1361-8415(01)00036-6

Niv, Y. (2009). Reinforcement learning in the brain. *Journal of Mathematical Psychology*, *53*(3), 139–154. https://doi.org/10.1016/j.jmp.2008.12.005

O'Doherty, J. P., Dayan, P., Friston, K., Critchley, H., & Dolan, R. J. (2003). Temporal Difference Models and Reward-Related Learning in the Human Brain. *Neuron*, *38*(2), 329–337. https://doi.org/10.1016/S0896-6273(03)00169-7

Patriat, R., Reynolds, R. C., & Birn, R. M. (2017). An improved model of motion-related signal changes in fMRI. *NeuroImage*, *144, Part A*, 74–82. https://doi.org/10.1016/j.neuroimage.2016.08.051

Pauli, W. M., Nili, A. N., & Tyszka, J. M. (2018). A high-resolution probabilistic in vivo atlas of human subcortical brain nuclei. *Scientific Data*, *5*(1), 180063. https://doi.org/10.1038/sdata.2018.63

Power, J. D., Mitra, A., Laumann, T. O., Snyder, A. Z., Schlaggar, B. L., & Petersen, S. E. (2014). Methods to detect, characterize, and remove motion artifact in resting state fmri. *NeuroImage*, *84*(Supplement C), 320–341. https://doi.org/10.1016/j.neuroimage.2013.08.048

Satterthwaite, T. D., Elliott, M. A., Gerraty, R. T., Ruparel, K., Loughead, J., Calkins, M. E., Eickhoff, S. B., Hakonarson, H., Gur, R. C., Gur, R. E., & Wolf, D. H. (2013). An improved framework for confound regression and filtering for control of motion artifact in the preprocessing of resting-

state functional connectivity data. *NeuroImage*, *64*(1), 240–256. https://doi.org/10.1016/j.neuroimage.2012.08.052

Schultz, W. (2024). A dopamine mechanism for reward maximization. *Proceedings of the National Academy of Sciences*, *121*(20), e2316658121. https://doi.org/10.1073/pnas.2316658121

Schwarzkopf, D. S. (2016, October). Example Prf Data Set For Samsrf. https://doi.org/10.5281/ZENODO.163582

Tustison, N. J., Avants, B. B., Cook, P. A., Zheng, Y., Egan, A., Yushkevich, P. A., & Gee, J. C. (2010). N4itk: Improved n3 bias correction. *IEEE Transactions on Medical Imaging*, *29*(6), 1310–1320. https://doi.org/10.1109/TMI.2010.2046908

Wilson, R. C., & Collins, A. G. (2019). Ten simple rules for the computational modeling of behavioral data. *eLife*, *8*, e49547. https://doi.org/10.7554/eLife.49547

Worthy, D. A., Otto, A. R., Cornwall, A. C., Don, H. J., & Davis, T. (2018). A Case of Divergent Predictions Made by Delta and Decay Rule Learning Models. *CogSci ... Annual Conference of the Cognitive Science Society. Cognitive Science Society (U.S.). Conference*, *2018*, 1175–1180.

Zeidman, P., Silson, E. H., Schwarzkopf, D. S., Baker, C. I., & Penny, W. (2018). Bayesian population receptive field modelling. *NeuroImage*, *180*, 173–187. https://doi.org/10.1016/j.neuroimage.2017.09.008

Zhang, Y., Brady, M., & Smith, S. (2001). Segmentation of brain MR images through a hidden markov random field model and the expectation-maximization algorithm. *IEEE Transactions on Medical Imaging*, *20*(1), 45–57. https://doi.org/10.1109/42.906424
